# Assessment of antibacterial and anti-biofilm activities of marine fungal epiphytes associated with red and green algae of the Kenyan coast

**DOI:** 10.1016/j.biotno.2026.06.003

**Published:** 2026-06-24

**Authors:** Aragaw Zemene Sendekie, Kimang'a Andrew Nyerere, Purity Kinya Kaaria

**Affiliations:** a Department of Molecular Biology and Biotechnology, Pan African University Institute for Basic Sciences, Technology and Innovation (PAUSTI), P. O. Box: 62000-00200, Nairobi, Kenya; b Department of Medical Biotechnology, Institute of Biotechnology (IoB), University of Gondar, P. O. Box: 196, Gondar, Ethiopia; c Department of Medical Microbiology, College of Health Sciences, Jomo Kenyatta University of Agriculture and Technology (JKUAT), P. O. Box: 62000-00200, Nairobi, Kenya; d Department of Botany, School of Biological Sciences, Jomo Kenyatta University of Agriculture and Technology (JKUAT), P. O. Box: 62000-00200, Nairobi, Kenya

**Keywords:** Antibacterial, Anti-biofilm, Epiphytic fungi, GC-MS analysis, SEM imaging

## Abstract

Marine-derived fungal epiphytes represent an important yet largely unexplored source of bioactive compounds. This study investigates the antibacterial and anti-biofilm properties of cultivable fungal epiphytes associated with red and green algae collected from a single coastal site in Kenya. A total of 330 fungal isolates were initially identified based on their morphological characteristics. Following agar-plug screening, nine active isolates have been identified through ITS-rDNA sequencing. Ethyl acetate and methanolic fungal extracts were evaluated against six multidrug-resistant microorganisms using disc diffusion, MIC, MBC, and microtiter biofilm disruption assays. The five best-performing extracts were subjected to SEM imaging, with two (Cer sp-2 and Ulr-1) further analyzed via GC-MS. Extracts from both solvents showed remarkable antibacterial effects, producing zones of inhibition that spanned between 10.00 ± 0.00 and 29.00 ± 0.00 mm. Furthermore, MIC and MBC assays revealed strong activity, with the lowest recorded values being 0.039 mg/mL and 0.156 mg/mL, respectively. Scanning electron microscopy (SEM) analysis displayed structural changes in bacterial cells, supporting a membrane-targeting mechanism of action. They also exhibited significant anti-biofilm properties (P < 0.05-0.0001) compared to the PBS-treated control. Although most biofilm reduction percentages were relatively low, the extracts displayed measurable activity in disrupting pre-formed biofilms. GC-MS identified a diverse profile of bioactive metabolites in the two representative extracts. Overall, Kenyan coastal algae harbor bioactive fungal epiphytes with promising potential to combat multidrug-resistant and biofilm-forming pathogens. Comprehensive studies are needed to discover novel therapeutic candidates in the future.

## 1 Introduction

For several centuries, nature has functioned as a rich reservoir of therapeutic agents, providing humanity with a sustainable source of structurally diverse natural products.^1^ A significant proportion of modern drugs trace their origins to bioactive compounds derived from plants, microorganisms, and marine organisms, many of which were initially identified through traditional medicinal practices.^2^ Although considerable progress has been made in terrestrial bioprospecting, marine ecosystems still remain a relatively untapped frontier for novel drug discovery. Covering more than 70% of the Earth's surface, the oceans harbor extraordinary biodiversity and chemical richness, yielding over 15,000 unique natural compounds from marine organisms.^3^ This immense diversity offers vast potential for discovering new molecular scaffolds with pharmaceutical value, particularly from marine microorganisms.^4^

Among marine-derived microbes, fungi have emerged as a dominant and prolific source of secondary metabolites with wide-ranging biological activities. More than 60% of investigated marine microbial strains, including those associated with mangroves, sponges, and algae, are fungi.^5^ These marine fungi have attracted growing attention for producing unique chemical entities belonging to the alkaloids, terpenoids, peptides, and polyketides, many of which exhibit antimicrobial, cytotoxic, antioxidant, anticancer, and anti-biofilm properties.^6^ Their metabolic plasticity and ability to thrive under fluctuating salinity, temperature, and nutrient conditions often lead to the biosynthesis of novel compounds that are rare or absent in terrestrial species.^7^

Marine macroalgae, classified as green, brown, and red seaweeds, are critical coastal ecosystem components that act as primary producers and ecological niches for diverse symbiotic marine life, including bacteria, actinomycetes, and fungi.^8^ Epiphytic and endophytic fungi inhabit on the external surfaces and internal tissues or even in the cellular structures of their hosts, respectively. In recent years, these host-associated marine fungi have received greater attention than free-living marine fungi from natural product chemists in the search for novel antimicrobial and other pharmaceutical properties.^9^ Bugni and Ireland reported that, among the 272 new compounds identified from marine-derived fungi till 2002, over 85% were produced by epiphytes and endophytes isolated from various marine environments.^10^

Despite the growing recognition of seaweed-associated fungi as a source of bioactive metabolites, their diversity and functional roles remain significantly underexplored in several tropical marine ecosystems, particularly along the Western Indian Ocean. The Kenyan coast, part of this biogeographically rich region, is home to a wide range of red and green macroalgae.^11^ Endophytes from *Gracilaria* species (red algae), for instance, produce bioactive compounds such as agarans, phenolic acids, diterpenes, and bromophenols with demonstrated antimicrobial, antioxidant, and cytotoxic activities.^12^ Similarly, fungal endophytes associated with *Ulva* species (green algae) are recognized for their production of sulfated polysaccharides, polyphenols, and terpenoids that contribute to their nutritional and therapeutic properties.^13^ Understanding these seaweed-associated fungal communities is essential, as they may play critical roles in algal defense mechanisms and serve as untapped sources of novel bioactive metabolites.^14^

The increasing prevalence of antibiotic-resistant pathogens has intensified the search for novel antimicrobial agents from natural sources.^15^ Marine-derived epiphytic fungi represent an underexploited resource for such bioactive molecules, especially along the East African coastline.^5^ Unlike endophytes, epiphytic fungi inhabit the algal surface where they are exposed to intense ecological pressures, including competition, ultraviolet radiation, salinity fluctuations, and host-derived chemical defenses.^16^ These conditions are likely to drive the production of unique adaptive metabolites with potent biological activities. Moreover, most previous studies in the region have primarily emphasized antibacterial activity, with limited attention to antibiofilm properties, which are crucial in combating persistent and drug-resistant infections. Thus, systematic exploration of marine fungal biodiversity, specifically those associated with red and green seaweeds, could produce new antibacterial leads of pharmaceutical relevance.^17^

Therefore, the present study focuses on the isolation and characterization of epiphytic fungi associated with red and green seaweeds collected from Kenyan coastal site within specific ecological settings. Using an integrated morpho-molecular approach, the study identifies cultivable fungal taxa and evaluates the antibacterial and anti-biofilm potential of their crude extracts against ESKAPE bacteria. Through this, the findings provide scientific insight into the potential of fungal epiphytes as promising sources of novel therapeutic compounds.

## 2 Materials and methods

### 2.1 Study area description

**Fig. 1:**
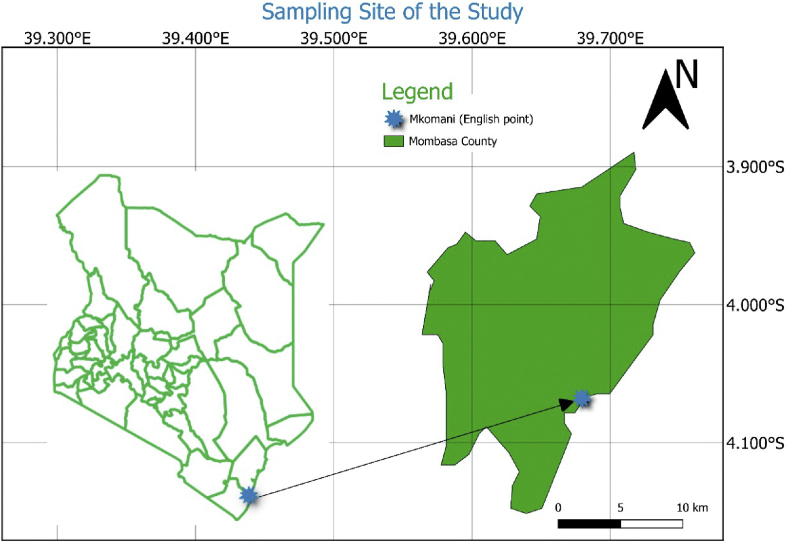
Map illustrating the sampling site for marine algae (seaweeds) collection along the Kenyan coast.

The study was conducted at Mkomani, a coastal site situated at approximately 4° 4′ South latitude and 39° 41′ East longitude (Fig. 1). This locality along the Kenyan coastline in the Western Indian Ocean has been surveyed for marine algae. The coastal region experiences two distinct monsoon seasons: the northeast monsoon (NM), locally known as “*kaskazi*”, and the southeast monsoon (SM), referred to as “*kusi*”. The SM occurs from May to September, whereas the NM prevails from November through March. In between these monsoons, there is a one-to two-month of transition period characterized by variable and relatively weak winds locally termed as “*matlai*”.^18^ These two seasons experience variations in atmospheric pressure, humidity, wind patterns, cloud cover, rainfall, evaporation, and solar radiation, collectively shaping the physicochemical and biological conditions of coastal oceanographic waters.^19^

### 2.2 Collection of seaweed samples and preparation

A total of 20 mature and healthy algal species were collected. These comprised 10 red and 10 green algae from the intertidal zones of the study site during spring-low tides in June 2024. The algal samples were carefully handpicked and rinsed with seawater to remove residual sand and undesirable debris. To maintain their physiological integrity and prevent desiccation, each sample was placed in a labeled, sterile zip-lock bag filled with seawater. The specimens were immediately transported to the Kenya Marine and Fisheries Research Institute (KMFRI) laboratory in ice-packed cool boxes. This ensured they remained in optimal condition for sorting and identification (Table 1).

**Table 1:** List of seaweeds taxonomically identified and authenticated from the coast of Kenya.

Algae/Seaweed samples collected				
No.	Red	Given code	Green	Given code
1	*Acanthophora spicifera*	R1/6/20/2024	*Cladophora* sp.	G1/6/20/2024
2	*Chondrophycus papillosus*	R2/6/20/2024	*Caulerpa scalpelliformis*	G2/6/20/2024
3	*Gracilariasalicornia*	R3/6/20/2024	*Ulva lactuca*	G3/6/20/2024
4	*Gracilaria corticata*	R4/6/20/2024	*Ulva fasciata*	G4/6/20/2024
5	*Hypnea musciformis*	R5/6/20/2024	*Halimeda macroloba*	G5/6/20/2024
6	*Hypnea pannosa*	R6/6/20/2024	*Ulva reticulata*	G6/6/20/2024
7	*Ceramium* sp.	R7/6/20/2024	*Boergesenia forbesii*	G7/6/20/2024
8	*Hypnea nidifica*	R8/6/20/2024	*Boodlea composita*	G8/6/20/2024
9	*Chondrophycus* sp.	R9/6/20/2024	*Caulerpa sertularioides*	G9/6/20/2024
10	*Botryocladia leptopoda*	R10/6/20/2024	*Cladophoropsis* sp.	G10/6/20/2024

Key: R-Red algae; G-Green algae.

### 2.3 Fungal epiphytes isolation and preservation

To isolate epiphytic fungi, freshly collected seaweeds underwent a sequential cleaning process. This included an initial double-rinse with sterile seawater followed by 10 min of gentle agitation (60 rpm) with sterile glass beads in flasks to remove surface-bound organisms.^20^ After a final twice-rinse in sterile seawater to remove any remaining transient or free-living fungal cells, the treated thalli were gently rubbed and inoculated onto Potato Dextrose Agar (PDA) enriched with 1 mg/mL chloramphenicol to suppress bacterial growth.^21^ The PDA was prepared with filtered and autoclaved seawater to simulate natural marine conditions and promote optimal fungal growth. Following inoculation, the plates were maintained under aerobic conditions at 28 ± 2 °C for 7–10 days. Distinct fungal phenotypes were then refined into pure cultures through multiple rounds of subculturing. These isolates were preserved on PDA slants and in potato dextrose broth (PDB) supplemented with 15% glycerol at 4 °C until needed for further tests.^22^

### 2.4 Agar-plug diffusion assay (primary screening)

The pure isolates of epiphytic fungi were initially screened for antibacterial activity using the agar-plug diffusion method.^23^ The lawns of test bacterial pathogens belonging to the ESKAPE group: *Enterococcus faecium*, *Staphylococcus aureus*, *Klebsiella pneumoniae*, *Acinetobacter baumannii*, *Pseudomonas aeruginosa*, and *Enterobacter cloacae* were prepared on Mueller-Hinton Agar (MHA) plates. Approval for the use of these clinically archived bacterial isolates was obtained from Mount Kenya University Research and Ethics Committee under approval number 4605. The study involved previously preserved clinical isolates and did not include direct human participation or access to patient-identifiable information. Agar plugs (6 mm in diameter) were aseptically cut from actively growing fungal cultures on PDA plates (not enriched with chloramphenicol) using a sterile cork-borer. These plugs were carefully placed on the surfaces of MHA plates seeded with test microorganisms (bacterial suspension adjusted to 0.5 McFarland standard) in triplicate.^24^ The plates were then kept at 4 °C overnight to allow diffusion of fungal metabolites, followed by incubation at 37 °C for 16–18 h to promote bacterial growth.^25^^,^^26^ The fungal isolates exhibiting clear zones of inhibition against at least one of the test pathogens were considered promising and selected for secondary screening.^26^ Broad-spectrum antibacterial activity was defined as the ability of an isolate to inhibit the growth of at least three tested bacterial pathogens, including both Gram-negative and Gram-positive types.

### 2.5 Phenotypic identification of fungal epiphytes

Morphological characterization remains one of the oldest and reliable approaches for the identification of microorganisms.^27^ In this study, nine selected fungal epiphytes showing bioactivity were identified based on their macroscopic and microscopic features. The cultural characteristics such as colony color and surface appearance (granular, powdery, mucoid, or smooth), texture, growth period, reverse color, and other features, were appropriately recorded.^28^ For microscopic examination, hyphae from the pure cultures were mounted on sterile slides, stained with lactophenol cotton blue solution, and observed under a light microscope (OPTIKA, Italy). The morphology of mycelia and conidiophores was observed and compared with previous descriptions.^29^ The identification process was further verified using standard taxonomic keys and reference descriptions given by,^30^ ensuring accurate and reliable phenotypic characterization. For convenience, the isolates were designated as: Acs-2, Cer sp-2, Hym-1, Hyp-5, Ula-1, Ula-9, Ulr-1, Ulr-18, and Ulr-22, where the numerals correspond to their respective colony numbers.

### 2.6 Molecular characterization of fungal epiphytes

#### 2.6.1 Genomic DNA extraction

Molecular identification was performed on nine epiphytic fungal isolates, obtained from red (four isolates) and green (five isolates) seaweed samples. Approximately 100 mg of mycelial biomass from 7 to 10 days old pure cultures grown on PDA were carefully scraped from the culture plates using a sterile surgical blade. Genomic DNA (gDNA) was extracted using the Quick-DNA™ fungal/bacterial Miniprep kit (Zymo Research Corp., CA 92614, USA) as per the manufacturer's protocol. The yield and purity of the resulting gDNA were quantified via a NanoDrop spectrophotometer (Thermo Fisher Scientific, USA). The concentration was expressed in ng/μL, and the purity of DNA was determined using the A260/A280 absorbance ratio. The DNA quality and its integrity were further verified through electrophoresis on a 1% (w/v) agarose gel.

#### 2.6.2 PCR amplification of the ITS region and sequencing

The internal transcribed spacer (ITS) region of the fungal rDNA was amplified using genomic DNA as a template and the universal primers ITS1-F (5′-TCCGTAGGTGAACCTGCGG-3′) and ITS4 (5′-TCCTCCGCTTATTGATATGC-3′).^31^ Reactions were conducted in a Biometra TRIO thermocycler (Analytik Jena, Germany), with an initial denaturation at 94 °C for 10 min, followed by 34 cycles of denaturation at 94 °C for 70 s, annealing at 56 °C for 60 s, and extension at 68 °C for 60 s. A final extension step was performed at 68 °C for 5 min. The presence of PCR products was validated by using 1% agarose gel electrophoresis in 1×TAE buffer at 90 V for 1 h and visualized with Safe-Red staining. The PCR amplicons were stored at −20 °C prior to shipment for bidirectional sequencing by Macrogen Ltd. (Singapore). Finally, the acquired sequences were deposited in the NCBI database under specific accession numbers.

#### 2.6.3 Phylogenetic analysis based on ITS region sequences

Following the generation of consensus sequences in BioEdit software (version 7.0.5.2), the BLASTN program was employed to retrieve closely related nucleotide sequences at the National Center for Biotechnology Information (NCBI) database: https://www.ncbi.nlm.nih.gov/nucleotide/.^32^ These reference sequences were aggregated with our experimental data into a FASTA format for alignment via ClustalW program (http://www.clustal.org).^33^ A neighbor-joining tree of the aligned sequences was constructed using MEGA11 software.^34^ Evolutionary distances were computed using the maximum composite Likelihood method.^35^ Statistical support value for the resulting clades was provided by 1000 bootstrap replicates.^36^ Any positions containing gaps were excluded pairwise from the analysis. This phylogenetic approach allowed for the definitive taxonomic assignment of each isolate to its respective group.

### 2.7 Culture of epiphytic fungi and extraction of secondary metabolites

Based on the primary screening of potent antibacterial activity, nine epiphytic isolates were selected for further small-scale production of secondary metabolites. The extraction of these metabolites followed the method described by Nurunnabi et al.^37^. A plug of 7-day-old mycelium (6 mm × 6 mm) from each isolate was inoculated into 250 mL conical flasks containing 100 mL of seawater potato dextrose broth (PDB). To stimulate biomass accumulation and metabolite secretion, the cultures were allowed to grow for three weeks on an orbital shaker at 120 rpm. PDB without fungal plug inoculation was also incubated under similar conditions and used as a sterility control to monitor potential contamination during the fermentation process of secondary metabolites extraction. Upon completion of the three-week incubation, the culture broth was separated from the mycelium via vacuum filtration using Whatman® Grade 1qualitative filter paper (Sigma-Aldrich, USA), and the filtrates were extracted three times with an equal volume of ethyl acetate (EtOAc) in a separating funnel. The mycelium was macerated in methanol under dark conditions for two days, then separated through filtration. Both the ethyl acetate (EtOAc) and methanolic fractions were concentrated to dryness at 40–45 °C using a rotary evaporator. The crude extracts were weighed and reconstituted in dimethyl sulfoxide (DMSO) to achieve a concentration of 20 mg/mL.^38^ These extract solutions were stored at −20 °C for subsequent bioassays.

### 2.8 Antibacterial testing using paper disc diffusion method

The antimicrobial activity of ethyl acetate and methanolic crude extracts from seaweed-associated fungal epiphytes was evaluated against a panel of multidrug-resistant (MDR) ESKAPE pathogens. The bacterial repertoire included Gram-negative (*Klebsiella pneumoniae*, *Acinetobacter baumannii*, *Pseudomonas aeruginosa*, and *Enterobacter cloacae*) and Gram-positive (*Enterococcus faecium* and *Staphylococcus aureus*) strains. Using the disc diffusion assay, as explained in the previous study,^39^ mid-log phase cultures were standardized to a 0.5 McFarland turbidity (1.5 × 10^8^ colony forming units (CFU/mL)) and uniformly inoculated onto Mueller-Hinton Agar (MHA) plates. Sterile 6 mm discs were impregnated with 20 μL of extract (20 mg/mL) and strategically positioned to prevent zone overlap. Plates were sealed with parafilm, kept in a refrigerator at 4 °C for 4 h, and then incubated at 37 °C for 24 h. Antibacterial effect was then quantified by measuring the diameter of inhibition zones (mm).^40^ To validate the reproducibility of the test, *Escherichia coli* (ATCC 25922) and *Staphylococcus aureus* (ATCC 25923) were used as reference strains. Experiments were performed in triplicate, with chloramphenicol and DMSO serving as positive and negative controls, respectively.

### 2.9 Minimum inhibitory concentration (MIC)

The minimum inhibitory concentration (MIC) of the ethyl acetate, methanolic, and combined crude extracts of epiphytic fungi was assessed against drug-resistant ESKAPE pathogens employed in the present study. The MIC was performed using a modified broth microdilution assay, adapted from the National Committee for Clinical Laboratory Standards (NCCLS).^41^ Crude extracts were reconstituted in DMSO and subjected to two-fold serial dilutions in Mueller-Hinton Broth (MHB) to attain a concentration range of 10 mg/mL to 0.039 mg/mL. Briefly, 100 μL of MHB was dispensed into each well of a 96-well microtiter plate. Subsequently, 100 μL of the stock extract was added to the first column and serially diluted across the plate to column 10, with the final 100 μL aliquot discarded. The bacterial turbidity was adjusted to a 0.5 McFarland standard in MHB. Each well (columns 1-11) was then inoculated with 100 μL of the bacterial suspension. Column 11 served as a positive growth control (broth and bacteria, no extract), while column 12 remained uninoculated as a negative sterility control (broth only). Following incubation at 37 °C for 18 h, the plates were inspected for visible turbidity. The MIC was defined as the lowest concentration of the extract that didn't allow observable bacterial growth. All assays were conducted in triplicate to ensure reproducibility.

### 2.10 Minimum bactericidal concentration (MBC)

Once the MIC values were determined, the minimum bactericidal concentration (MBC) of the ethyl acetate, methanolic, and combined extracts was also evaluated to evaluate their bactericidal potential. A volume of 10 μL aliquots was withdrawn from all wells exhibiting no turbidity and sub-cultured onto the surface of Mueller-Hinton Agar (MHA) plates. These plates were incubated at 37 °C for 24 h. The MBC was recorded as the lowest extract concentration that resulted in no visible bacterial growth on the petri dishes.^42^

### 2.11 Scanning electron microscopy (SEM) analysis

To investigate the morphological changes induced by the fungal metabolites, scanning electron microscopy (SEM) analysis was performed on representative Gram-positive (*Staphylococcus aureus*) and Gram-negative (*Enterobacter cloacae)* pathogens. Extracts from the five epiphytic fungal isolates (Acs-2, Cer sp-2, Ula-1, Ulr-1, and Ulr-18) which exhibited the best inhibitory profiles, were selected for this analysis. These extracts were prepared as a dual combination (1:1 v/v) of ethyl acetate-methanol extracts.

#### 2.11.1 Sample preparation and fixation

Bacterial cultures in the mid-log growth phase (approximately 1 × 10^8^ CFU/mL) were treated with an equal volume of the respective extracts at a concentration of 2× MIC. After a 12 h incubation at 37 °C, the bacterial cells were collected via centrifugation at 4,000×*g* and washed thrice with phosphate-buffered saline (PBS). The resulting pellets were fixed in 2.5% glutaraldehyde (diluted in PBS) and maintained at 4 °C overnight to preserve cellular integrity.

#### 2.11.2 Dehydration and imaging

Once fixation was complete, the samples underwent a systematic dehydration process using serially graded ethanol concentrations (30%, 50%, 70%, 90%, and 100%). Each dehydration step involved a 15 min incubation period, followed by centrifugation at 4,000×*g*. The processed samples were thereafter air-dried overnight at ambient temperature and spread onto carbon adhesive tape. Finally, surface morphology and structural alterations were visualized using a scanning electron microscope (JCM-7000 NeoScope™, JEOL, Japan).^43^

### 2.12 Anti-biofilm assessment

A semi-quantitative analysis of the crystal violet staining technique was applied to assess the anti-biofilm potential of dually combined ethyl acetate-methanol extracts (1:1 v/v) from five epiphytic fungi (Acs-2, Cer sp-2, Ula-1, Ulr-1, and Ulr-18) against all the test bacteria used in the study. Bacterial suspensions at a mid-log phase were prepared in tryptic soy broth (TSB) supplemented with 1% glucose to a final concentration of 5 × 10^7^ CFU/mL and seeded into 96-well plates.^44^ The plates were statically incubated at 37 °C for 48 h to allow biofilm formation. After incubation, the TSB with floating cells was removed, and the wells were gently washed thrice with Phosphate-buffered saline (PBS). The adherent biofilms were then fixed with Neutral Buffered Formalin (NBF) for 20 min. Following fixation, the biofilms were again washed three times and treated with 50 μL of the extract at a concentration equivalent to ½ MIC for 12 h at room temperature. The PBS-treated wells served as negative controls. Then, the biofilms were stained with 1% crystal violet for 15 min, after the extracts were removed and washed. Subsequently, the crystal violet was removed, and biofilms were washed, and the stain embedded in the biofilms was dissolved with 95% ethanol (200 μL) for 15 min at room temperature with gentle shaking. Finally, 150 μL of the dissolved stain was transferred to a new plate, and absorbance was measured at OD570 nm using a microplate reader (BIOTEK elx800, USA).^45^ The percentage of biofilm reduction was calculated by the formula:

$$\text{Percent}\;\text{reduction}\;\text{of}\;\text{Biofilm}=\left[\frac{\left(\text{Control}\;\text{OD}\;570\;\text{nm}-\text{Test}\;\text{OD}570\;\text{nm}\right)}{\text{Control}\;\text{OD}570\;\text{nm}}\right]\times 100$$

The amount of biofilm reduction was measured by comparing the absorbance values of the bacteria treated with extracts versus untreated bacteria. Every experiment was performed in triplicate.

### 2.13 Gas chromatography-mass spectrometry (GC-MS)-based metabolites profiling

Gas chromatography coupled to mass spectrometry was used to analyze the metabolite profiles of the two most active extracts, Cer sp-2 and Ulr-1, derived from red and green algae, respectively. GC-MS-based characterization of bioactive compounds was performed using a Shimadzu (Kyoto, Japan) GC-MS QP-2010SE system. The method of analysis involves the separation of gaseous analytes within a stationary-phase-coated column, according to their boiling points and chemical affinities, followed by subsequent ionization and fragmentation patterns of the analytes according to their mass-to-charge (*m/z*) ratios.^46^ Sample preparation was done based on the method of Cheseto et al.,^47^ with minor modifications. To prepare the samples, 1000 μL of the appropriate solvent (methanol for methanolic and ethyl acetate for ethyl acetate extracts) was added to the respective fungal metabolites. Then 100 mg of Na_2_SO_4_ was added to the samples for dehydration. The samples were vortex-mixed for 1 min, ultrasonicated (Branson 2510) for 10 min, and centrifuged for 5 min at 13,000 rpm (5 °C). After further filtration through glass wool, the supernatant was diluted, and a 1.0 μL aliquot was injected into the GC-MS system for analysis. The system consisted of a low-polarity BPX5 capillary column (30 m × 0.25 mm × 0.25 μm film thickness). The oven temperature started with 55 °C held for 1 min, then increased linearly at a rate of 10 °C/min until reaching a final temperature of 280 °C (run duration 30 min). Helium was used as a mobile phase at a rate of 1.08 mL/min. The samples were diluted to 1% (v/v) and injected using an AS3000 autosampler with a split ratio of 10:1 at 250 °C. The ion source and interface temperatures of the mass detector were set to 200 °C and 250 °C, respectively. Electrospray Ionization Mass Spectroscopy (ESI MS) was done at 70 eV in full scanning mode within 35-550 *m/z* range. Metabolites detected were then analyzed quantitatively based on the relative peak areas and qualitatively using the National Institute of Standards and Technology (NIST) Spectral Library.

### 2.14 Graphing and statistical analysis

All assays were conducted in triplicate, and the results were reported as mean ± standard deviation (SD). Differences among groups were assessed by one-way ANOVA followed by Tukey's post-hoc test for all pairwise comparisons. GraphPad Prism version 10 (GraphPad Software Inc., San Diego, CA, USA) was employed for graphing and data analysis. Differences in the mean values among several groups at ∗p < 0.05, ∗∗p < 0.01, ∗∗∗p < 0.001, ∗∗∗∗p < 0.0001 were considered statistically significant.

## 3 Results

### 3.1 Agar-plug diffusion assay (primary screening)

The results revealed that fungal epiphytes isolated from both red and green algae exhibited a varying level of antibacterial activity against the multidrug-resistant ESKAPE pathogens. In the primary agar-plug diffusion screening, a total of 330 fungal isolates obtained from 20 species of marine algae (10 red and 10 green) were assessed for their antibacterial potential. Among the red algae-associated fungi (n = 128), 14 isolates (10.94%) showed inhibitory effects on at least one of the test bacteria, while four isolates (3.13%) displayed broad-spectrum antibacterial activity by effectively inhibiting three or more bacterial pathogens used in the study. Similarly, from the green algal sources (n = 202), 31 isolates (15.35%) demonstrated inhibitory activities against at least one of the test bacteria. However, only five isolates (2.48%) showed broad-spectrum activity.

Altogether, these nine broadly active isolates (4 from red and 5 from green algae) with distinct cultural features were therefore selected for further investigations, including morpho-molecular identification, secondary antibacterial assay, and determination of MIC and MBC values. Interestingly, a total of five isolates, two from red algae (1.56%) and three from green algae (1.49%), exhibited strong inhibitory effects against all the tested bacterial pathogens, indicating they are promising candidates for further bioprospecting. Table 2, Table 3 summarize these results, illustrating the distribution and strength of antibacterial activities among the isolates.

**Table 2:** Agar plug-based screening of antibacterial activity in epiphytic fungi from red algae.

Host red algae and their assigned codes	Total no. of isolates per host	No. of active isolates	No. of isolates with broad- spectrum activity	No. of isolates active against all test bacteria
*Acanthophora spicifera* (*Acs*)	12	3	1	1
*Chondrophycus papillosus (Chp)*	10	1	-	-
*Gracilaria Salicornia* (*Grs*)	9	-	-	-
*Gracilaria corticata* (*Grc*)	6	-	-	-
*Hypnea musciformis* (*Hym*)	24	2	1	-
*Hypnea pannosa* (*Hyp*)	15	3	1	-
*Ceramium* sp. (*Cer* sp.)	13	1	-	1
*Hypnea nidifica* (*Hyn*)	11	1	1	-
*Chondrophycus* sp. (*Cho* sp.)	16	2	-	-
*Botryocldia leptopoda* (*Bol*)	12	1	-	-

Total	**128**	**14**	**4**	**2**

Key: Isolate codes were given based on the scientific name of their host. (−) denotes no detectable activity.

**Table 3:** Agar plug-based screening of antibacterial activity in epiphytic fungi from green algae.

Host green algae and their assigned codes	Total no. of isolates per host	No. of active isolates	No. of isolates with broad- spectrum activity	No. of isolates active against all test bacteria
*Cladophora* sp. (*Cla* sp.)	11	4	-	-
*Caulerpa scalpelliformis* (*Casc*)	16	1	-	-
*Ulva lactuca* (*Ula*)	22	2	2	1
*Ulva fasciata* (*Ulf*)	20	5	-	-
*Halimeda macroloba* (*Ham*)	28	2	-	-
*Ulva reticulate* (*Ulr*)	27	7	3	2
*Boergesenia forbesii* (*Bof*)	14	6	-	-
*Boodlea composita* (*Boc*)	20	4	-	-
*Caulerpa sertularioides* (*Case*)	25	-	-	-
*Cladophoropsis* sp. (*Cla* sp.)	19	-	-	-

Total	**202**	**31**	**5**	**3**

Key: Isolate codes were given based on the scientific name of their host. (−) denotes no detectable activity.

### 3.2 Phenotypic identification of fungal epiphytes

In this study, the isolated pure cultures of fungal epiphytes were cultured on petri dishes and left to grow for 7–10 days to reach a stage of maturity. During this incubation period, the outer structure, colors of the mycelium and spores, as well as the pattern of mycelium formation, were observed through morphological evaluation. Morphological identification of fungi usually involves both macroscopic (colonies) and microscopic observations. Macroscopic evaluation involves observing the appearance of the colony, the texture of mycelia, and pigmentation on the surface/reverse of the culture plate, whereas microscopic observation involves the arrangements of conidia, conidiophore, and branching pattern.^48^ Epiphytic fungi isolated from red and green algae showed distinct colony appearance and pigmentation. Based on bioactivity, nine potent fungal isolates (4 from red and 5 from green algae) were morphologically identified and compared with reference species (Table 4).

**Table 4:** Morphological profiles of fungal epiphytes isolated from red and green macroalgae following 7-10 days of incubation on PDA medium at 28 ± 2 °C.

Code of isolates	Macroscopic characterization			Microscopic characterization		Putative epiphytic fungus
	Color of colony (front/reverse)	Texture	Shape	Mycelium characters	Conidia/Spore Features	
Acs-2	White to pinkish/Reddish to brown	Fast-growing, fluffy to powdery	Circular	Cottony/floccose aerial, septate, and hyaline	Macroconidia: sickle-shaped, 3–5 septa; Microconidia: oval, single or in chains	*Fusarium* sp-1^49^
Cer sp-2	Dark green/slight yellowish to brown	Powdery, dense and fast growth	Circular	Velvety, compact, septate (cross-walls) and transparent	Round, greenish, finely rough-walled conidia	*Aspergillus* sp-2^51^
Hym-1	White to yellowish brown/Orange-brown	Fast-growing, rough edges	Circular	Cottony and floccose aerial mycelium	Macroconidia: falcate, with 3–5 septa; Microconidia: oval to cylindrical	*Fusarium* sp-2^28^
Hyp-5	Blue-green/Yellowish-brown	Velvety, compact growth	Circular	Fine, globose asci with interwoven mycelium	Round to elliptical conidia in chains	*Talaromyces* sp-1^51^
Ula-1	White to off-white/Light yellowish	Cottony and fast growing	Circular, concentric ring structure	Likely septate, cottony and transparent	Macroconidia slightly curved, 3–5 septate; Microconidia oval	*Fusarium* sp-3^28^
Ula-9	Dark olive-brown/Black	Velvety, moderate growth	Circular	Septate, dark-pigmented	Conidia obclavate, multiple septa	*Alternaria* sp-1^50^
Ulr-1	Gray-green to light yellow/Yellow-brown	Powdery, compact	Circular	Fine, septate, dense and inconspicuous	Small conidia in chains	*Penicillium* sp-1^52^
Ulr-18	Dark brown to black/light brown	Suede-like, slow-growing	Circular	Compact, septate, dark-pigmented	Spherical, black conidia, echinulate surface	*Aspergillus* sp-1^51^
Ulr-22	Dark brown to black/Black	Suede-like, moderate growth	Irregular	Septate, dark-pigmented	Large, obclavate conidia with multiple septa	*Alternaria* sp-2^50^

**Fig. 2:**
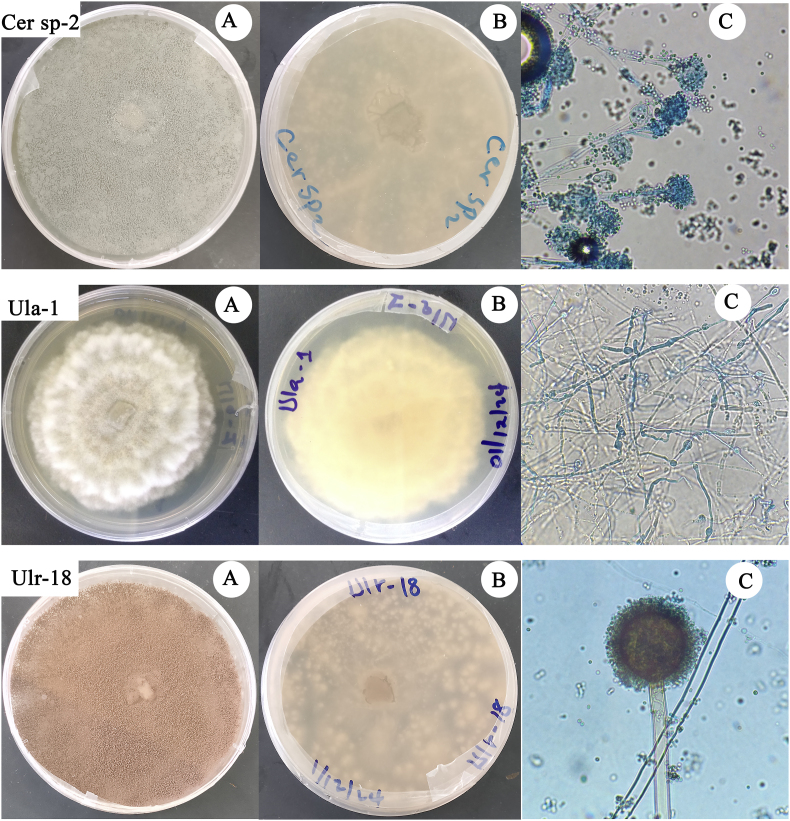

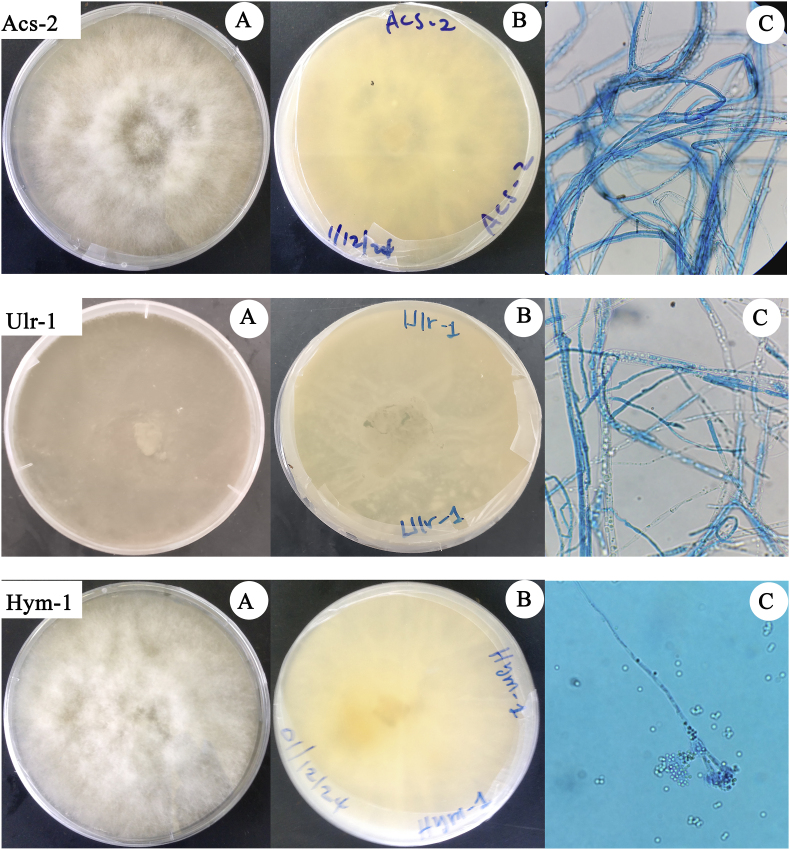

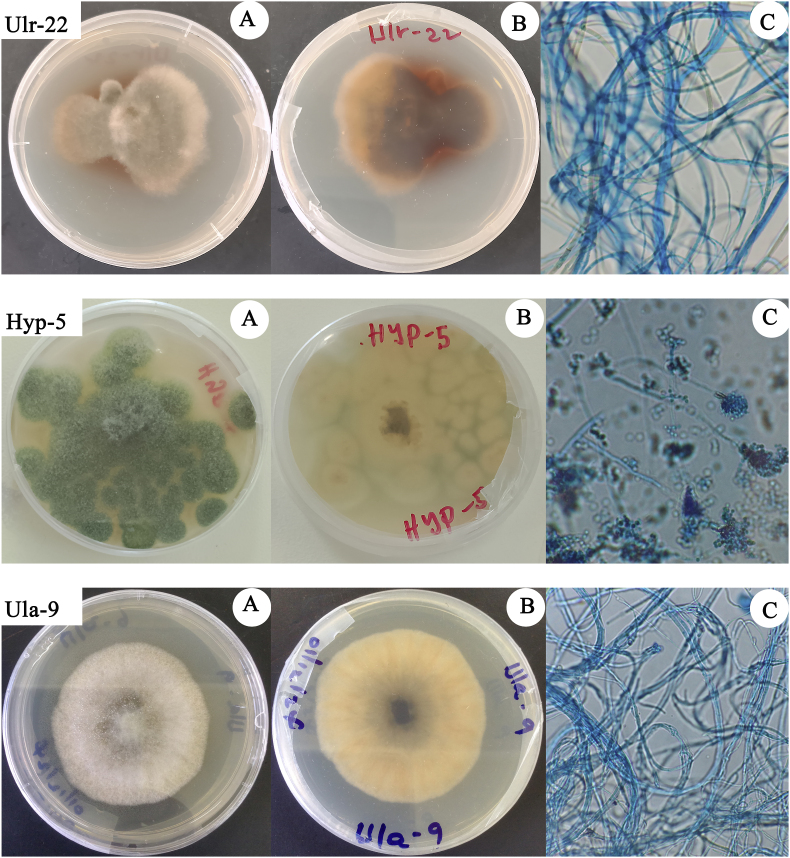
Morphological characterization of epiphytic fungal isolates. Fungal cultures were grown on PDA for 7-10 days, at 28 ± 2 °C. Front view (**A**), Reverse view (**B**), Microscopic view (**C**). Magnification of each slide was 40x by observing the shape of conidia and hyphae. The identification process was guided by the established taxonomic keys and descriptive literature.^30^

The phenotypic characteristics of the identified genera were presented in Fig. 2 and further described in Table 4. The isolate Acs-2 corresponding to *Fusarium* sp-1 developed cottony, white to pinkish colonies with a reddish-brown reverse, and produced sickle-shaped, multi-septate macroconidia.^49^ The isolate Hym-1, which morphologically resembled *Fusarium* sp-2, had floccose white to yellowish brown colonies with fusiform macroconidia containing 3-5 septa. The isolate Ula-1 was distinguished by a concentric growth pattern with white to off-white mycelia on the surface and light-yellow pigmentation on the reverse side of the culture. Such morphological attributes indicate a strong similarity with *Fusarium* sp-3.^28^ Among the *Alternaria* isolates, *Alternaria* sp-1 (Ula-9) produced dark olive-brown colonies with septate, pigmented mycelium. *Alternaria* sp-2 (Ulr-22) was identified by its larger and deeply septate conidia.^50^ Among the *Aspergillus* isolates, isolate Cer sp-2 formed dark green, powdery colonies with a yellowish reverse. The conidiophores were unbranched, ending in a globose vesicle, and bore conidia arranged in linear chains. The conidia were round and pale green in color. The colony morphology and microscopic observations suggested the identity of the isolate Cer sp-2 resembled a defining characteristic of *A. fumigatus,* which was later confirmed by the nucleotide BLAST report of ITS sequences.^51^ The isolate Ulr-18 was also identified by suede-like, dark brown to black colonies and echinulate, spherical conidia. According to the observed features, the isolate Ulr-18 was identified as *Aspergillus* sp-1. *Talaromyces* sp-1 (isolate Hyp-5) was identified based on its biverticillate conidiophores and globose conidia. The colonies of Hyp-5 appeared compact with a characteristic blue-green coloration.^51^ Finally, isolate Ulr-1 (*Penicillium* sp-1) was characterized by its branched and smooth-walled conidiophores with small conidia in chains.^52^

Fungal epiphytes with a relatively distinct morphological features were identified based on their macroscopic and microscopic observations. The mycelium and the spores were stained with lactophenol cotton blue for microscopic identification purposes.

### 3.3 Molecular characterization and phylogeny

**Fig. 3:**
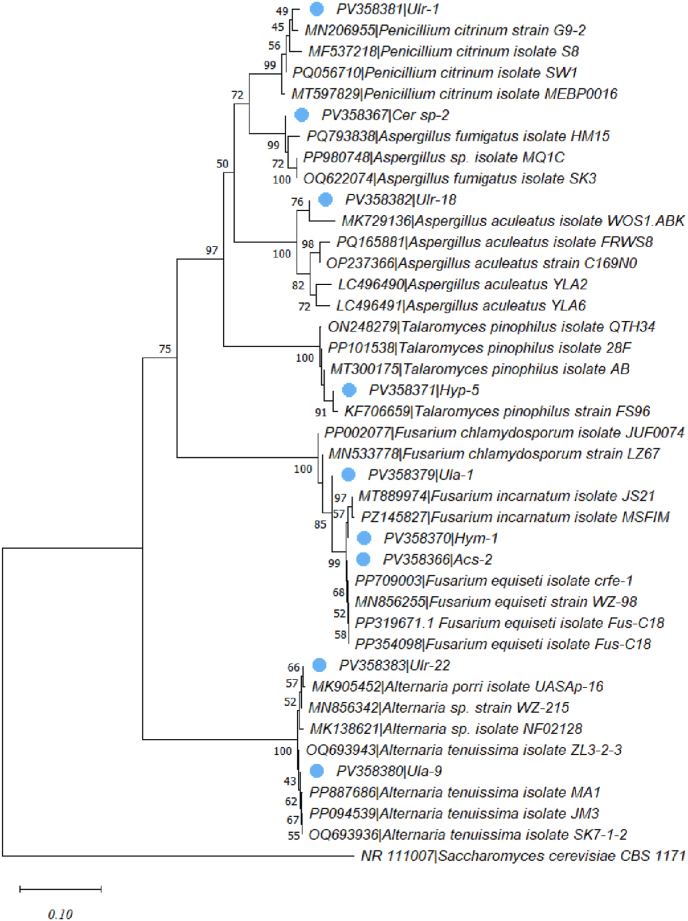
Phylogenetic tree of nine fungal isolates showing their genetic relationships based on ITS sequence data.

To elucidate the evolutionary relationships of the isolated fungal epiphytes, ITS-based phylogenetic tree was constructed using MEGA11. The phylogenetic analysis revealed that all isolates belong to the phylum Ascomycota, with the resulting topology partitioning into five distinct genera: *Penicillium*, *Aspergillus*, *Talaromyces*, *Alternaria*, and *Fusarium*. These genera were distributed across three families: *Trichocomaceae* (*Penicillium*, *Aspergillus*, and *Talaromyces*), *Pleosporaceae* (*Alternaria*), and *Nectriaceae* (*Fusarium*). Each isolate clustered closely with known reference sequences retrieved from GenBank, thereby confirming their taxonomic identities and providing strong molecular support for the primary morphological characterizations (Fig. 3).

As presented in Fig. 3 and Table 5, the nucleotide BLAST analysis of isolate Acs-2 (Accession no. PV358366) showed 99.82% sequence similarity to its best hit with 100% query coverage. It had the closest evolutionary relationship with the subclade *Fusarium equiseti* (PP709003), supported by a bootstrap value of 99%. The isolate Cer sp-2 (Accession no. PV358367) exhibited a 100% sequence similarity and was phylogenetically closely related to the subclade *Aspergillus fumigatus* (PQ793838) with strong bootstrap support (91%). The isolate Hym-1 (Accession no. PV358370) revealed a phylogenetic relationship (bootstrap value of 57% and identity 99.81%) with the GenBank ITS sequence of *Fusarium incarnatum* (PZ145827). The isolate Hyp-5 (Accession no. PV358371) showed the closest similarity to the GenBank of *Talaromyces pinophilus* (KF706659) with a strong bootstrap value of 91% and 99.62% identity. The isolate Ula-1 (Accession no. PV358379) was placed in the *Fusarium* clade, with high affinity to the GenBank ITS sequence of *Fusarium chlamydosporum* (MN533778), supported by a bootstrap 100% and 99.80% similarity. The nucleotide BLAST result of isolate Ula-9 (Accession no. PV358380) grouped within the *Alternaria* clade and was closest to the GenBank sequence of *Alternaria tenuissima* (PP887686) with a bootstrap value of 62% and 100% similarity. Similarly, isolate Ulr-22 (Accession no. PV358383) was positioned within the *Alternaria* genus, specifically with *Alternaria porri* (MK905452), showing 99.30% identity and 66% of bootstrap value. The isolate Ulr-1 (Accession no. PV358381) indicated evolutionary similarity with *Penicillium citrinum* (MN20695), supported by a weak bootstrap 49% and 98.69% of identity. The isolate Ulr-18 (Accession no. PV358382) was clustered within *Aspergillus*, closest to *Aspergillus aculeatus* (MK729136) with a relatively strong bootstrap value of 76% and 96.71% of phylogenetic similarity. From an overall perspective, the ITS phylogeny resolved most of the isolates into well-supported clades, and the closest sequence matches were consistent with their cultural and microscopic morphology.

**Table 5:** BLAST matches for fungal epiphytes from red and green seaweeds of the Kenyan coast.

Sample ID	Host seaweed species	Accession Number	Closest taxonomic affiliation from BLAST search	% Query cover	% Identity
Acs-2	*Acanthophora spicifera*	PV358366	*Fusarium equiseti PP709003*	100	99.82
Cer sp-2	*Ceramium* sp.	PV358367	*Aspergillus fumigatus* PQ793838	100	100.00
Hym-1	*Hypnea musciformis*	PV358370	*Fusarium incarnatum* PZ145827	100	99.81
Hyp-5	*Hypnea pannosa*	PV358371	*Talaromyces pinophilus* KF706659	100	99.62
Ula-1	*Ulva lactuca*	PV358379	*Fusarium chlamydosporum* MN533778	100	99.80
Ula-9	*Ulva lactuca*	PV358380	*Alternaria tenuissima* PP887686	100	100.00
Ulr-1	*Ulva reticulate*	PV358381	*Penicillium citrinum* MN206955	100	98.69
Ulr-18	*Ulva reticulate*	PV358382	*Aspergillus aculeatus* MK729136	100	96.71
Ulr-22	*Ulva reticulate*	PV358383	*Alternaria porri* MK905452	100	99.30

BLASTN analysis confirmed that the sequences of the fungal epiphytes shared 96.71–100% identity with their respective reference sequences in the GenBank (Table 5). These identified rDNA-ITS sequences have been deposited in GenBank database for public access.

The phylogenetic analyses were performed using MEGA11.^35^ The evolutionary relationships were inferred using the Neighbor-Joining method based on ITS rDNA sequences, encompassing the ITS1, 5.8S, and ITS4 regions.^53^ In the phylogenetic tree, epiphytic fungal isolates obtained from the red and green seaweeds of the present study were designated by green circles (Fig. 3). The reliability of the tree topology was evaluated using bootstrap analysis with 1000 replicates; the percentage of trees in which associated taxa clustered together was indicated next to the corresponding branches.^36^ Evolutionary distances were calculated using the p-distance method, expressed as the number of base substitutions per site. To refine the dataset, all ambiguous positions were excluded for each sequence pair via the pairwise deletion option.

### 3.4 Antibacterial testing using paper disc diffusion method

After primary antibacterial screening, the crude extracts from selected fungal epiphytes that showed promising bioactive performance against multidrug-resistant ESKAPE pathogens were subjected to further evaluation using disc diffusion assay. For comparison, DMSO and chloramphenicol were used as negative and positive controls, respectively. The antibacterial potential of ethyl acetate and methanolic crude extracts was then assessed against drug-resistant ESKAPE microorganisms, and the mean inhibition zones were presented in Table 6, Table 7.

**Table 6:** Antibacterial activity of ethyl acetate crude extracts against drug-resistant ESKAPE pathogens and susceptible reference strains.

Types of Extracts	Mean zone of inhibition (mm) ± Standard deviation							
	*E. coli* ATCC 25922	*S. aureus* ATCC 25923	*E. cloacae*	*A. baumannii*	*E. faecium*	*S. aureus*	*K. pneumoniae*	*P. aeruginosa*
Acs-2	18.75 ± 0.48**^b^**	15.43 ± 0.93**^de^**	13.77 ± 0.93**^bc^**	12.67 ± 1.15**^c^**	16.00 ± 1.00**^bc^**	10.83 ± 0.76**^f^**	10.23 ± 0.15**^e^**	15.67 ± 0.58**^de^**
Cer sp-2	24.80 ± 0.62**^a^**	27.65 ± 0.12**^a^**	22.33 ± 0.58**^a^**	23.17 ± 0.29**^a^**	26.67 ± 0.58**^a^**	27.33 ± 0.58**^a^**	14.80 ± 0.35**^b^**	29.00 ± 0.00**^a^**
Hym-1	13.30 ± 0.66**^d^**	14.55 ± 1.87**^e^**	0.00 ± 0.00**^d^**	0.00 ± 0.00**^d^**	13.00 ± 0.00**^d^**	12.27 ± 0.25**^ef^**	11.50 ± 0.50**^d^**	15.83 ± 0.76**^d^**
Hyp-5	15.60 ± 0.26**^cd^**	15.11 ± 0.16**^de^**	12.13 ± 0.12**^c^**	0.00 ± 0.00**^d^**	14.08 ± 0.88**^cd^**	13.17 ± 0.29**^e^**	12.41 ± 0.37**^cd^**	17.33 ± 0.58**^c^**
Ula-1	15.52 ± 1.10**^cd^**	17.22 ± 0.30**^cd^**	12.83 ± 0.29**^c^**	14.33 ± 0.58**^bc^**	13.87 ± 0.76**^cd^**	15.67 ± 0.58**^d^**	13.57 ± 0.32**^bc^**	14.33 ± 0.29**^ef^**
Ula-9	17.56 ± 1.15**^bc^**	11.13 ± 1.18**^f^**	13.00 ± 0.00**^c^**	15.50 ± 0.87**^b^**	16.80 ± 1.21**^b^**	0.00 ± 0.00**^g^**	12.53 ± 0.42**^cd^**	13.20 ± 0.20**^fg^**
Ulr-1	26.99 ± 1.00**^a^**	28.37 ± 0.49**^a^**	15.40 ± 0.36**^b^**	23.42 ± 0.52**^a^**	24.50 ± 1.00**^a^**	25.17 ± 1.04**^b^**	16.33 ± 0.58**^a^**	26.60 ± 0.35**^b^**
Ulr-18	17.43 ± 1.17**^bc^**	18.35 ± 1.38**^c^**	12.90 ± 1.28**^c^**	13.43 ± 0.51**^c^**	17.23 ± 1.37**^b^**	18.97 ± 0.95**^c^**	12.50 ± 0.87**^cd^**	14.10 ± 0.52**^f^**
Ulr-22	14.81 ± 0.92**^d^**	18.12 ± 0.69**^c^**	13.50 ± 0.50**^c^**	13.00 ± 1.00**^c^**	17.00 ± 1.00**^b^**	16.83 ± 0.29**^d^**	0.00 ± 0.00**^f^**	12.27 ± 0.64**^g^**
CHL	26.50 ± 0.00**^a^**	24.17 ± 0.00**^b^**	0.00 ± 0.00**^d^**	0.00 ± 0.00**^d^**	0.00 ± 0.00**^e^**	0.00 ± 0.00**^g^**	0.00 ± 0.00**^f^**	0.00 ± 0.00**^h^**
DMSO	0.00 ± 0.00**^e^**	0.00 ± 0.00**^g^**	0.00 ± 0.00**^d^**	0.00 ± 0.00**^d^**	0.00 ± 0.00**^e^**	0.00 ± 0.00**^g^**	0.00 ± 0.00**^f^**	0.00 ± 0.00**^h^**

∗Values were reported as Mean ± standard deviation (SD) of triplicates; Means with different superscripts along a column were statistically different p < 0.05 (Tukey's post-hoc test); DMSO: negative control; CHL (chloramphenicol): positive control.

**Table 7:** Antibacterial activity of methanolic crude extracts against drug-resistant ESKAPE pathogens and susceptible reference strains.

Types of Extracts	Mean zone of inhibition (mm) ± Standard deviation							
	*E. coli* ATCC 25922	*S. aureus* ATCC 25923	*E. cloacae*	*A. baumannii*	*E. faecium*	*S. aureus*	*K. pneumoniae*	*P. aeruginosa*
Acs-2	15.20 ± 0.63**^c^**	12.72 ± 0.53**^e^**	11.50 ± 0.50**^cde^**	10.67 ± 1.15**^d^**	13.00 ± 1.00**^de^**	10.23 ± 0.40**^f^**	10.00 ± 0.00**^e^**	13.50 ± 0.50**^de^**
Cer sp-2	21.63 ± 1.10**^b^**	29.64 ± 0.48**^a^**	20.33 ± 0.58**^a^**	21.13 ± 0.23**^a^**	24.67 ± 0.58**^a^**	28.00 ± 1.00**^a^**	13.78 ± 0.60**^ab^**	29.00 ± 0.00**^a^**
Hym-1	11.20 ± 0.84**^d^**	12.79 ± 0.66**^e^**	10.23 ± 0.25**^e^**	0.00 ± 0.00**^e^**	11.10 ± 0.17**^e^**	11.23 ± 0.25**^ef^**	10.17 ± 0.29**^e^**	13.33 ± 0.58**^de^**
Hyp-5	14.35 ± 0.39**^c^**	13.24 ± 0.13**^e^**	10.67 ± 1.15**^de^**	0.00 ± 0.00**^e^**	13.00 ± 0.00**^de^**	12.00 ± 1.00**^e^**	10.67 ± 1.15**^e^**	15.67 ± 0.58**^c^**
Ula-1	13.93 ± 0.23**^c^**	16.10 ± 0.11**^d^**	12.00 ± 0.00**^cde^**	13.5 ± 0.61**^bc^**	12.83 ± 0.76**^de^**	14.77 ± 0.40**^d^**	12.93 ± 0.12**^bc^**	14.00 ± 0.00**^d^**
Ula-9	15.21 ± 0.58**^c^**	10.78 ± 0.55**^f^**	12.40 ± 0.17**^cd^**	14.67 ± 1.15**^b^**	15.17 ± 1.04**^cd^**	0.00 ± 0.00**^g^**	12.00 ± 0.00**^cd^**	12.33 ± 0.58**^ef^**
Ulr-1	21.28 ± 0.96**^b^**	25.15 ± 0.31**^b^**	14.57 ± 0.12**^b^**	21.33 ± 0.58**^a^**	22.00 ± 1.00**^b^**	24.67 ± 0.58**^b^**	15.38 ± 0.54**^a^**	25.67 ± 0.58**^b^**
Ulr-18	15.16 ± 0.39**^c^**	18.00 ± 0.00**^c^**	11.67 ± 1.53**^cde^**	11.67 ± 0.58**^cd^**	15.33 ± 1.53**^cd^**	17.90 ± 0.17**^c^**	10.70 ± 1.13**^de^**	13.48 ± 0.45**^de^**
Ulr-22	13.90 ± 0.82**^c^**	17.33 ± 0.42**^cd^**	13.30 ± 0.79**^bc^**	12.00 ± 1.00**^cd^**	16.00 ± 1.00**^c^**	16.00 ± 0.00**^d^**	0.00 ± 0.00**^f^**	11.90 ± 0.17**^f^**
CHL	26.50 ± 0.00**^a^**	24.17 ± 0.00**^b^**	0.00 ± 0.00**^f^**	0.00 ± 0.00**^e^**	0.00 ± 0.00**^f^**	0.00 ± 0.00**^g^**	0.00 ± 0.00**^f^**	0.00 ± 0.00**^g^**
DMSO	0.00 ± 0.00**^e^**	0.00 ± 0.00**^g^**	0.00 ± 0.00**^f^**	0.00 ± 0.00**^e^**	0.00 ± 0.00**^f^**	0.00 ± 0.00**^g^**	0.00 ± 0.00**^f^**	0.00 ± 0.00**^g^**

∗Values were reported as Mean ± standard deviation (SD) of triplicates; Means with different superscripts within a column were statistically different p < 0.05 (Tukey's post-hoc test); DMSO: negative control; CHL (chloramphenicol): positive control.

The antibacterial activity of the ethyl acetate crude extracts was evaluated against multidrug-resistant ESKAPE pathogens, as well as susceptible reference strains of *E. coli* ATCC 25922 and *S. aureus* ATCC 25923 (Table 6). The significance of each group was represented by superscript letters (a–h). The distinct letters indicate that their results are statistically different, and any observed difference between them is considered to be significant (p < 0.05). The fungal extracts demonstrated variable inhibitory effects, with zones of inhibition ranging from no activity to as high as 29.00 ± 0.00 mm. The extracts Acs-2, Cer sp-2, Ula-1, Ulr-1, and Ulr-18 displayed antibacterial properties across all tested bacteria, characterized by broad-spectrum activity against both Gram-positive and Gram-negative bacteria. Among the extracts, Cer sp-2 and Ulr-1 exhibited the strongest inhibitory activity, showing high inhibition against both resistant ESKAPE pathogens and the reference strains. Notably, Cer sp-2 displayed the highest inhibition zones, ranging from 14.80 ± 0.35 mm (*K. pneumoniae*) to 29.00 ± 0.00 (*P. aeruginosa*), while Ulr-1 exhibited inhibition zones between 15.40 ± 0.36 mm (*E. cloacae*) and 28.37 ± 0.49 mm (*S. aureus* ATCC 25923). Intermediate antibacterial potential was also observed in the remaining extracts. They presented inhibition zones ranging from 10.23 ± 0.15 mm (Acs-2 against *K. pneumonia*) to 18.97 ± 0.95 mm (Ulr-18 against *S. aureus*). However, Ulr-22, Hyp-5, Ula-9, and Hym-1 showed no detectable antibacterial properties against some of the ESKAPE pathogens. There was no zone of inhibition observed in the negative control (DMSO), indicating that the solvent used to dissolve the extracts does not affect the test pathogens. Furthermore, the positive control (CHL) exhibited a strong zone of inhibition against the susceptible reference strains but was not effective against the resistant microorganisms. In contrast, the fungal extracts showed intermediate to strong activity against both standard and resistant bacteria, although some extracts displayed selective or no activity against specific microorganisms (Table 6).

As shown in Table 7, a significant difference (P < 0.05) in the diameter of the inhibition zones was observed when the test bacteria were treated with methanolic extracts. The significance of each group was expressed in superscripts (a-g). Methanolic extracts of Acs-2, Cer sp-2, Ula-1, Ulr-1, and Ulr-18 were also exhibited antibacterial activity against the entire panel of tested strains, demonstrating a broad-spectrum inhibitory profile. The maximum inhibition was observed with Cer sp-2 [diameter 13.78 ± 0.60 mm (*K. pneumonia*) to 29.64 ± 0.48 mm (*S. aureus* ATCC 25923)] and Ulr-1 [diameter 14.57 ± 0.12 mm (*E. cloacae*) to 25.67 ± 0.58 mm (*P. aeruginosa*)] against the tested bacteria. Measurable inhibitory activity was observed in Ula-1, Ula-9, Ulr-18, and Ulr-22 extracts, which presented inhibition zones in the range of approximately 10.78-18.00 mm. However, methanolic extracts of Acs-2, Hym-1, and Hyp-5 (except *P. aeruginosa*) displayed relatively weaker inhibitory activity. Extracts Ulr-22, Hyp-5, Ula-9, and Hym-1 showed no detectable antibacterial properties against some of the test pathogens, reflecting selectivity in antibacterial potential. Notably, the methanolic extracts exhibited inhibitory activity against resistant pathogens that were remained non-susceptible to the conventional antibiotics used as a control (Table 7).

MIC and MBC data for the fungal crude extracts against ESKAPE pathogens were summarized in Table 8. MIC values spanned 0.039–2.5 mg/mL and MBC values 0.156–5.0 mg/mL, depending on the property of the isolate and target organism. Extracts: Acs-2, Cer sp-2, Ula-1, Ulr-1, and Ulr-18 were persistently the most active across all test bacteria. Remarkably, Cer sp-2 showed the lowest MIC (0.039 mg/mL) against *P. aeruginosa*, followed by low MICs (0.078 mg/mL) against *E. faecium* and *S. aureus*, and by Ulr-1 against *P. aeruginosa*. In many cases, the MBC/MIC ratio was ≤4, indicating a predominantly bactericidal profile for the most active extracts. Conversely, Hym-1 (against *E. cloacae* and *A. baumannii*), Hyp-5 (against *A. baumannii*), Ula-9 (against *S. aureus*), and Ulr-22 (against *K. pneumonia*) were inactive and showed no detectable activity under the test conditions.

**Table 8:** Determination of the MIC and MBC (mg/mL) of the ethyl acetate extracts of fungal epiphytes against the tested bacteria.

Types of Extracts	Test ESKAPE microorganisms											
	*E. cloacae*		*A. baumannii*		*E. faecium*		*S. aureus*		*K. pneumoniae*		*P. aeruginosa*	
	MIC (mg/mL)	MBC (mg/mL)	MIC (mg/mL)	MBC (mg/mL)	MIC (mg/mL)	MBC (mg/mL)	MIC (mg/mL)	MBC (mg/mL)	MIC (mg/mL)	MBC (mg/mL)	MIC (mg/mL)	MBC (mg/mL)
Acs-2	1.25	2.5	2.5	5.0	1.25	2.5	2.5	2.5	2.5	5.0	1.25	2.5
Cer sp-2	0.156	0.3125	0.156	0.3125	0.078	0.156	0.078	0.156	1.25	2.5	0.039	0.156
Hym-1	-	-	-	-	2.5	5.0	2.5	5.0	2.5	5.0	1.25	2.5
Hyp-5	2.5	5.0	-	-	1.25	2.5	1.25	2.5	2.5	5.0	0.625	1.25
Ula-1	2.5	2.5	1.25	2.5	1.25	2.5	1.25	2.5	1.25	5.0	1.25	2.5
Ula-9	2.5	5.0	1.25	2.5	0.625	2.5	-	-	2.5	5.0	1.25	2.5
Ulr-1	1.25	2.5	0.156	0.3125	0.156	0.625	0.156	0.625	1.25	2.5	0.078	0.3125
Ulr-18	2.5	5.0	1.25	5.0	0.625	1.25	0.625	0.3125	2.5	5.0	1.25	2.5
Ulr-22	2.5	2.5	2.5	2.5	0.625	2.5	0.625	2.5	-	-	2.5	2.5

(−) indicates no activity observed, while MIC and MBC represent Minimum inhibitory concentration and Minimum bactericidal concentration, respectively. All ethyl acetate extracts of fungal epiphytes were tested in triplicates, and the mean MIC values were determined.

The minimum inhibitory and bactericidal activities of fungal extracts against ESKAPE pathogens were summarized in Table 9. The MIC values ranged from 0.039 to 2.5 mg/mL, while MBC values extended from 0.156 to 5.0 mg/mL, indicating variability in potency across isolates and target pathogens. Five extracts, namely, Acs-2, Cer sp-2, Ula-1, Ulr-1, and Ulr-18, were active against all test bacteria. Cer sp-2 consistently demonstrated the strongest activity, with MICs as low as 0.039 mg/mL against *S. aureus* and *P. aeruginosa* and MBCs of 0.156 mg/mL, highlighting potent bactericidal potential. Similarly, Ulr-1 displayed very good antibacterial effects against *P. aeruginosa* (MIC 0.078 mg/mL, MBC 0.3125 mg/mL). Unlike its ethyl acetate counterpart, the methanol extract of Hym-1 showed an activity against *E. cloacae*, with MIC of 2.5 mg/mL and MBC of 5.0 mg/mL. However, Hym-1 (against *A. baumannii*), Hyp-5 (against *A. baumannii*), Ula-9 (against *S. aureus*), and Ulr-22 (against *K. pneumonia*) displayed no detectable activity during the test.

**Table 9:** Determination of the MIC and MBC (mg/mL) of the methanolic extracts of fungal epiphytes against the tested bacteria.

Types of Extracts	Test ESKAPE microorganisms											
	*E. cloacae*		*A. baumannii*		*E. faecium*		*S. aureus*		*K. pneumoniae*		*P. aeruginosa*	
	MIC (mg/mL)	MBC (mg/mL)	MIC (mg/mL)	MBC (mg/mL)	MIC (mg/mL)	MBC (mg/mL)	MIC (mg/mL)	MBC (mg/mL)	MIC (mg/mL)	MBC (mg/mL)	MIC (mg/mL)	MBC (mg/mL)
Acs-2	2.5	5.0	2.5	5.0	2.5	2.5	2.5	5.0	2.5	5.0	1.25	2.5
Cer sp-2	0.3125	0.625	0.3125	0.625	0.156	0.3125	0.039	0.156	1.25	2.5	0.039	0.156
Hym-1	2.5	5.0	-	-	2.5	5.0	2.5	2.5	2.5	5.0	2.5	2.5
Hyp-5	2.5	5.0	-	-	2.5	2.5	2.5	2.5	2.5	5.0	2.5	2.5
Ula-1	2.5	5.0	1.25	5.0	2.5	2.5	1.25	2.5	2.5	5.0	1.25	2.5
Ula-9	2.5	5.0	1.25	5.0	1.25	2.5	-	-	2.5	5.0	2.5	5.0
Ulr-1	1.25	2.5	0.3125	1.25	0.156	0.625	0.156	0.625	0.156	1.25	0.078	0.3125
Ulr-18	2.5	5.0	2.5	5.0	0.625	2.5	0.625	1.25	2.5	5.0	1.25	5.0
Ulr-22	2.5	5.0	2.5	5.0	1.25	5.0	1.25	2.5	-	-	2.5	5.0

(−) indicates no activity observed, while MIC and MBC represent Minimum inhibitory concentration and Minimum bactericidal concentration, respectively. All methanol extracts of fungal epiphytes were tested in triplicates, and the mean MIC values were determined.

The MIC and MBC of fungal crude extracts prepared with methanol and ethyl acetate (combined) against the test pathogens were presented in Table 10. Like their individual ethyl acetate and methanol counterparts, the combined (1:1 v/v) extracts of Acs-2, Cer sp-2, Ula-1, Ulr-1, and Ulr-18 were effective against all ESKAPE pathogens tested in the study. The MIC values ranged from 0.039 to 2.5 mg/mL, while MBC values varied between 0.078 and 5.0 mg/mL. This clearly reflects differences in potency across isolates and pathogens. Cer sp-2 exhibited the strongest, with remarkably lowest MIC and MBC values of 0.039 and 0.078 mg/mL, respectively, against *P. aeruginosa*, and equally low values of 0.078 mg/mL for both MIC and MBC against *E. faecium* and *S. aureus*. Likewise, Ulr-1 has also shown strong effects, with MICs of 0.039 and MBC of 0.156 mg/mL against *P. aeruginosa*, followed by MIC of 0.078 mg/mL and MBC of 0.3125 mg/mL against *A. baumannii*, *E. faecium*, and *S. aureus*. Nevertheless, Hym-1 (against *A. baumannii*), Hyp-5 (against *A. baumannii*), Ula-9 (against *S. aureus*), and Ulr-22 (against *K. pneumonia*) exhibited no measurable activity. The observed effects reflect preliminary combined activity and should not be interpreted as confirmed synergy.

**Table 10:** Determination of the MIC and MBC (mg/mL) of the combined extracts of fungal epiphytes against the tested bacteria.

Types of Extracts	Test ESKAPE microorganisms											
	*E. cloacae*		*A. baumannii*		*E. faecium*		*S. aureus*		*K. pneumoniae*		*P. aeruginosa*	
	MIC (mg/mL)	MBC (mg/mL)	MIC (mg/mL)	MBC (mg/mL)	MIC (mg/mL)	MBC (mg/mL)	MIC (mg/mL)	MBC (mg/mL)	MIC (mg/mL)	MBC (mg/mL)	MIC (mg/mL)	MBC (mg/mL)
Acs-2	1.25	1.25	1.25	1.25	0.625	0.625	2.5	2.5	2.5	2.5	0.625	0.625
Cer sp-2	0.156	0.156	0.156	0.156	0.078	0.078	0.078	0.078	1.25	1.25	0.039	0.078
Hym-1	2.5	5.0	-	-	1.25	2.5	2.5	2.5	1.25	2.5	1.25	2.5
Hyp-5	2.5	5.0	-	-	1.25	1.25	1.25	1.25	1.25	2.5	0.625	1.25
Ula-1	1.25	2.5	1.25	2.5	1.25	2.5	0.625	1.25	1.25	2.5	1.25	2.5
Ula-9	1.25	2.5	1.25	2.5	0.625	1.25	-	-	2.5	5.0	1.25	2.5
Ulr-1	1.25	2.5	0.078	0.3125	0.078	0.3125	0.078	0.3125	0.625	1.25	0.039	0.156
Ulr-18	1.25	2.5	1.25	2.5	0.3125	1.25	0.3125	1.25	1.25	2.5	1.25	2.5
Ulr-22	1.25	2.5	1.25	2.5	0.625	2.5	0.625	1.25	-	-	1.25	2.5

(−) indicates no activity observed, while MIC and MBC represent Minimum inhibitory concentration and Minimum bactericidal concentration, respectively. The combined extracts of fungal epiphytes were tested in triplicates, and the mean MIC values were determined.

Except for the methanolic extract of Ulr-1 against *S. aureus*, which exhibited a bacteriostatic effect, all other ethyl acetate, methanolic, and combined fungal extracts demonstrated bactericidal activity (MBC/MIC ≤4) against the tested bacteria (Table 11). Both ethyl acetate and methanolic extracts independently showed predominantly consistent bactericidal performance across the majority of fungal isolates, with only a few instances of no detectable activity. The combined extracts generally enhanced bactericidal efficacy, with isolates such as Acs-2, Cer sp-2, Ula-1, Ulr-1, and Ulr-18 exhibiting strong and consistent activity against all the microorganisms. Nevertheless, some combinations still remained inactive against specific pathogens tested in the present study.

**Table 11:** Summarizes the antibacterial index of ethyl acetate, methanolic, and combined fungal extracts against tested bacteria.

Types of Extracts	Antibacterial index (MBC/MIC Ratio): Ethyl acetate extract					
	*E. cloacae*	*A. baumannii*	*E. faecium*	*S. aureus*	*K. pneumoniae*	*P. aeruginosa*
	MBC/MIC	MBC/MIC	MBC/MIC	MBC/MIC	MBC/MIC	MBC/MIC
Acs-2	2	2	2	1	2	2
Cer sp-2	2	2	2	2	2	4
Hym-1	-	-	2	2	2	2
Hyp-5	2	-	2	2	2	2
Ula-1	1	2	2	2	4	2
Ula-9	2	2	4	-	2	2
Ulr-1	2	2	4	4	2	4
Ulr-18	2	4	2	2	2	2
Ulr-22	1	1	4	4	-	1

**Antibacterial index (MBC/MIC Ratio): Methanolic extract**						

Acs-2	2	2	1	2	2	2
Cer sp-2	2	2	2	4	2	4
Hym-1	2	-	2	1	2	1
Hyp-5	2	-	1	1	2	1
Ula-1	2	4	1	2	2	2
Ula-9	2	4	2	-	2	2
Ulr-1	2	4	4	4	8	4
Ulr-18	2	2	4	2	2	4
Ulr-22	2	2	4	2	-	2

**Antibacterial index (MBC/MIC Ratio): Combined extracts**						

Acs-2	1	1	1	1	1	1
Cer sp-2	1	1	1	1	1	2
Hym-1	2	-	2	1	2	2
Hyp-5	2	-	1	1	2	2
Ula-1	2	2	2	2	2	2
Ula-9	2	2	2	-	2	2
Ulr-1	2	4	4	4	2	4
Ulr-18	2	2	4	4	2	2
Ulr-22	2	2	4	2	-	2

(−) indicates no activity observed; MIC: Minimum inhibitory concentration; MBC: Minimum bactericidal concentration; MBC/MIC ≤4 is bactericidal (BC) and MBC/MIC *>* 4 is bacteriostatic (BS) effect.

### 3.5 Mode of action analysis

#### 3.5.1 Morphological changes of the bacterial cells after extract treatment

**Fig. 4:**
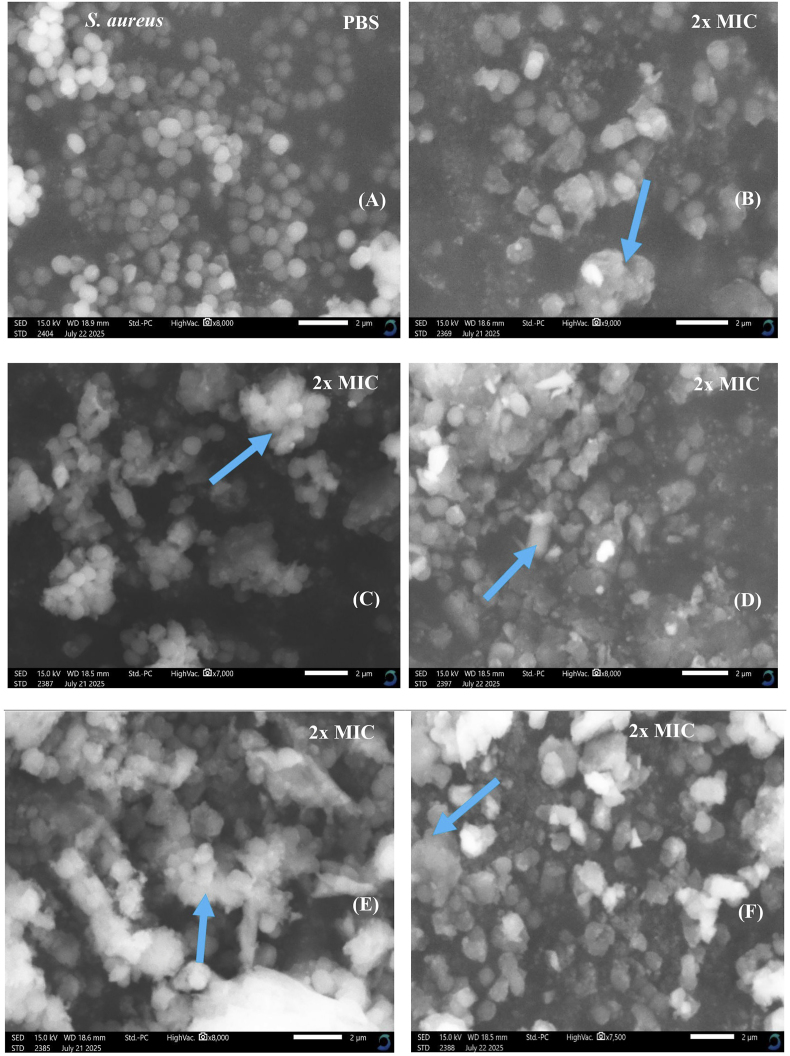
SEM images showing the effects of combined extracts at the concentration of 2x MIC mg/mL on membranes of *S. aureus* compared to the control. (A) 1x PBS-treated cells (control). (B–F) were extract-treated cells with Acs-2, Cer sp-2, Ula-1, Ulr-1, and Ulr-18, respectively. Arrows indicating representative damage.

**Fig. 5:**
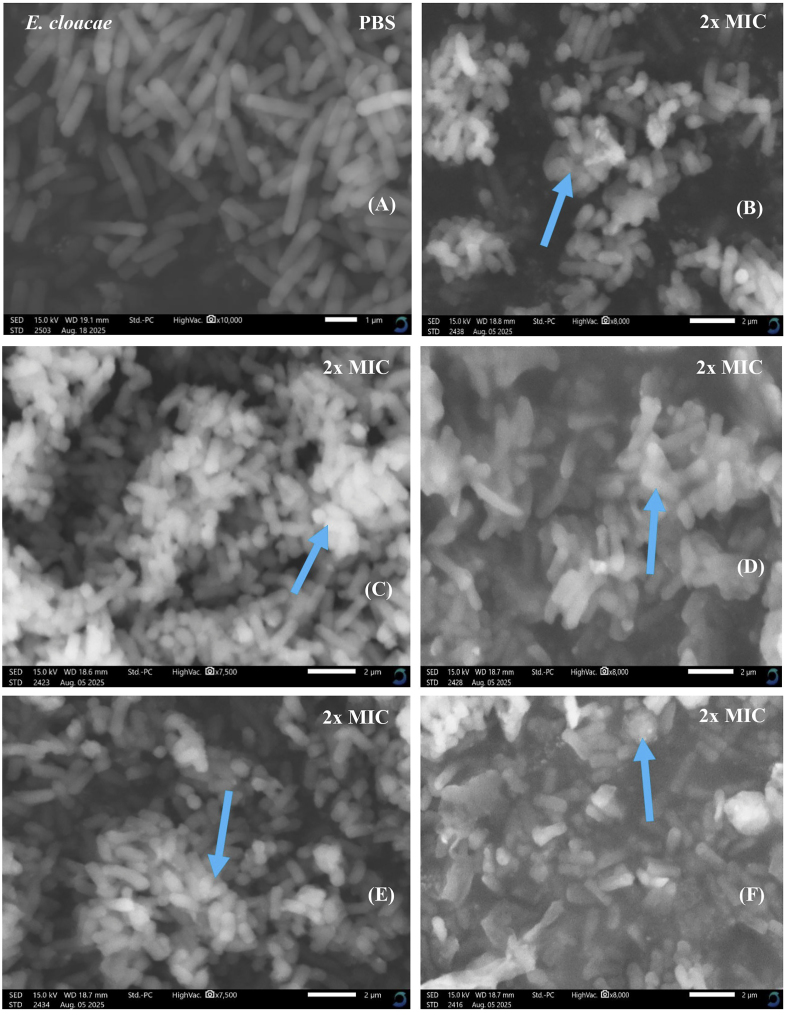
SEM micrographs shows how the combined fungal extracts at the concentration of 2x MIC mg/mL destroyed the membranes of *E. cloacae* compared to the control. (A) 1x PBS-treated cells (control). (B–F) were extract-treated cells with Acs-2, Cer sp-2, Ula-1, Ulr-1, and Ulr-18, respectively. Arrows indicating representative damage.

The effects of the combined (1:1 v/v) ethyl acetate and methanol extracts from isolates Acs-2, Cer sp-2, Ula-1, Ulr-1, and Ulr-18 on Gram-positive and Gram-negative bacteria were examined using SEM, with the resulting micrographs presented in Fig. 4, Fig. 5, respectively. Fig. 4, Fig. 5A were the controls (PBS-treated) cells of *S. aureus* and *E. cloacae*, respectively. The control *S. aureus* cells exhibited an intact, rigid coccoid morphology with smooth cellular surfaces (Fig. 4A). However, after 12 h of exposure to the extracts, the cells lost their intact spherical shape, underwent lysis, and shrank substantially (Fig. 4B–F).

Similarly, Fig. 5 illustrates the scanning electron micrographs of a control and treated Gram-negative bacterial cells, *E. cloacae*, before and after treatment with extracts. The controls (PBS-treated) cells existed in normal cell condition with typical rod shape, rigid, and smooth cell surface (Fig. 5A). However, after the cells were treated with the extracts for overnight, noticeable morphological alterations were observed, including cell surface invagination and cavity formation. These SEM analyses demonstrate that the extracts induced severe structural damage, eventually leading to the disruption of the cell membrane (Fig. 5B–F).

### 3.6 Evaluation of anti-biofilm activity

In addition to evaluating the antibacterial potential of the fungal extracts, the present study also investigated the anti-biofilm formation activity of the five best and broad-spectrum extracts—Acs-2, Cer sp-2, Ula-1, Ulr-1, and Ulr-18 against the test pathogens. It was found that a concentration of 5 × 10^7^ CFU/mL of the bacteria was the most suitable for the biofilm biomass formation. After 48 h of incubation, all of the test bacteria used in this study were able to form biofilm. The anti-biofilm assays were performed using sub-inhibitory (½ MIC) concentrations to specifically assess the ability of the extracts to interfere with biofilm development without exerting direct bactericidal effects. The anti-biofilm evaluation (OD at 570 nm) of the extracts against each of the test bacteria was presented in the following graphs.

**Fig. 6:**
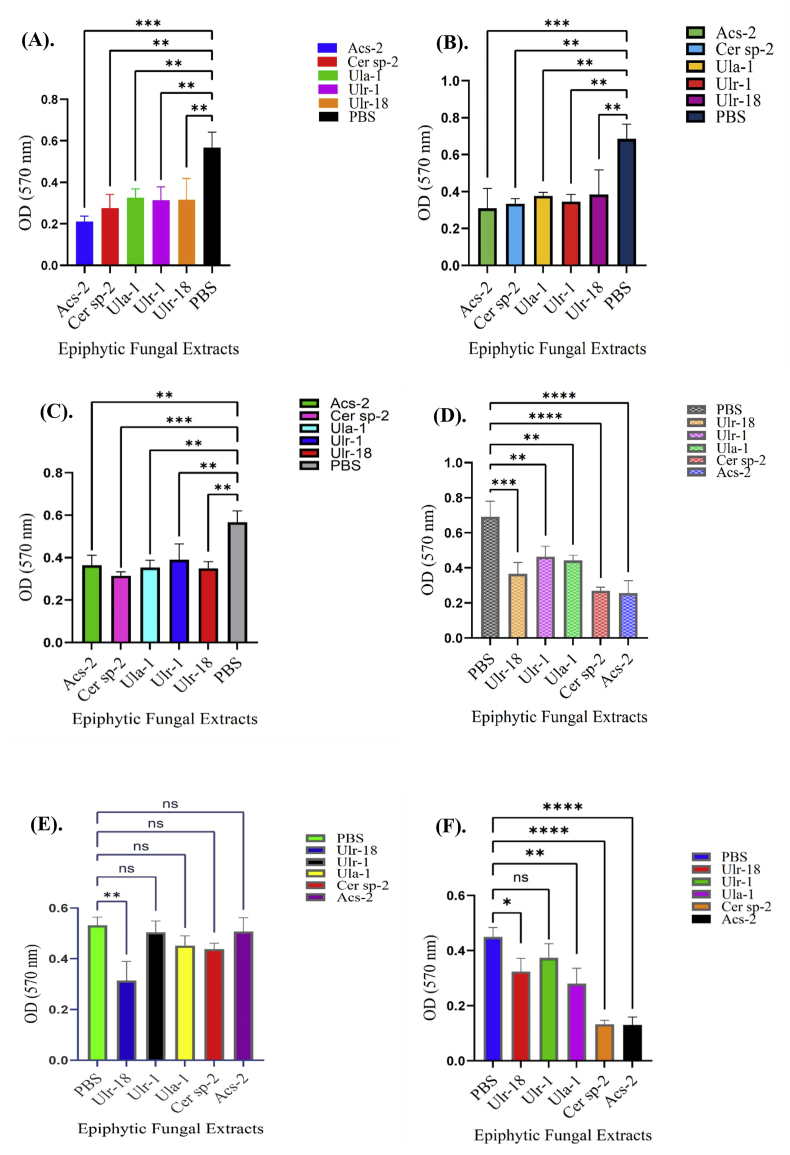
Anti-biofilm function of fungal extracts and the negative control against *E. cloacae***(A)**, *A. baumannii***(B)**, *E. faecium***(C)**, *S. aureus***(D)**, *K. pneumonia***(E)**, *P. aeruginosa***(F)**. The destruction of the biofilm strains was significantly different (∗p < 0.05, ∗∗P *<* 0.01, ∗∗∗P *<* 0.001, and ∗∗∗∗P *<* 0.0001) compared to the control (PBS).

Based on the analysis of the semi-quantitative crystal violet assay, all epiphytic fungal extracts displayed a reduction of biofilm development by the tested bacteria compared to the untreated control (PBS). The control group without extract (PBS) demonstrated the highest biofilm biomass formation, while treatment with fungal extracts led to a pronounced and statistically significant reduction (∗p < 0.05, ∗∗P *<* 0.01, ∗∗∗P *<* 0.001, and ∗∗∗∗P *<* 0.0001). Among the extracts, Acs-2 exhibited the best anti-biofilm effect against most of the tested pathogens, including *E. cloacae*, *A. baumannii*, *S. aureus*, and *P. aeruginosa*, as reflected by its lowest optical density at 570 nm. Fungal extracts from Cer sp-2 and Ulr-18 showed relatively higher anti-biofilm activity against *E. faecium* and *K. pneumoniae*, respectively. Ulr-1 was the only extract that didn't show a statistically significant anti-biofilm effect against *P. aeruginosa*. With the exception of Ulr-18, none of the other extracts exhibited a statistically significant reduction in biofilm formation activity by *K. pneumoniae* when compared to the control (Fig. 6A–F).

#### 3.6.1 Percent of biofilm reduction (%)

Table 12 shows the percentage of biofilm reduction, usually rated on a scale from 0 to 100%. Values between 0% and 50% indicate weak anti-biofilm activity, while percentages above 50% represent strong biofilm reduction. A reduction percentage below 0% is considered biofilm growth enhancement.^54^ All the extracts had various levels of biofilm reduction activity against the test bacteria. According to the results, only Acs-2 and Cer sp-2 extracts showed more than 50% biofilm reduction against *E. cloacae*, *A. baumannii*, *S. aureus*, and *P*. *aeruginosa*. On the other hand, there was no biofilm reduction of >50% of *E. faecium* and *K. pneumoniae* biofilm by all the fungal extracts. Except for Acs-2 and Cer sp-2, which showed good biofilm reduction activity, the rest of the extracts had moderately weak biofilm reduction activity (ranging from 5.08% to 49.56%) against the test bacteria used in the study.^45^^,^^54^

**Table 12:** Biofilm reduction percent of fungal extracts against ESKAPE bacteria.

Fungal Extracts	Biofilm reduction (%)					
	*E. cloacae*	*A. baumannii*	*E. faecium*	*S. aureus*	*K. pneumoniae*	*P. aeruginosa*
Acs-2	63.14	54.96	35.69	62.86	4.70	71.05
Cer sp-2	51.41	51.31	44.35	61.13	17.67	70.38
Ula-1	42.61	45.04	37.63	36.13	15.04	37.64
Ulr-1	44.72	49.56	30.92	33.09	5.08	16.70
Ulr-18	44.54	44.02	38.34	47.11	40.98	27.84

### 3.7 Gas chromatography-mass spectrometry (GC-MS) analysis

**Fig. 7:**
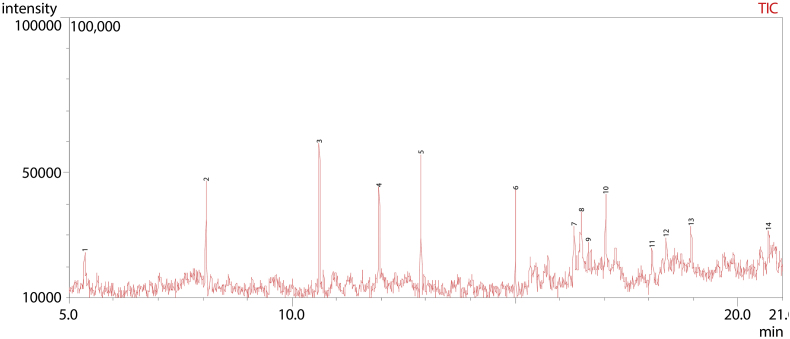
GC-MS chromatogram showing detected compounds in ethyl acetate extract of Cer sp-2. Numbers positioned over the peaks indicate the corresponding specific chemical components.

**Fig. 8:**
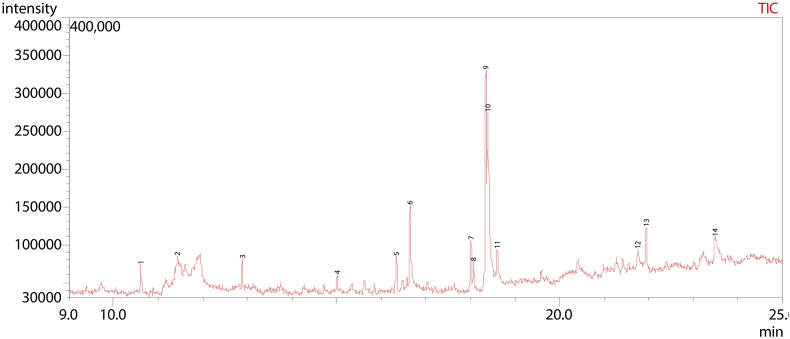
GC-MS chromatogram showing detected compounds in the methanolic extract of Cer sp-2. Numbers positioned over the peaks indicate the corresponding specific chemical components.

**Fig. 9:**
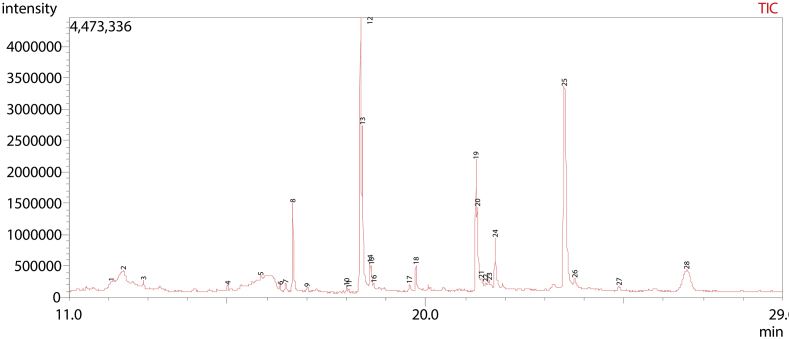
GC-MS chromatogram showing detected compounds in ethyl acetate extract of Ulr-1. Numbers positioned over the peaks indicate the corresponding specific chemical components.

**Fig. 10:**
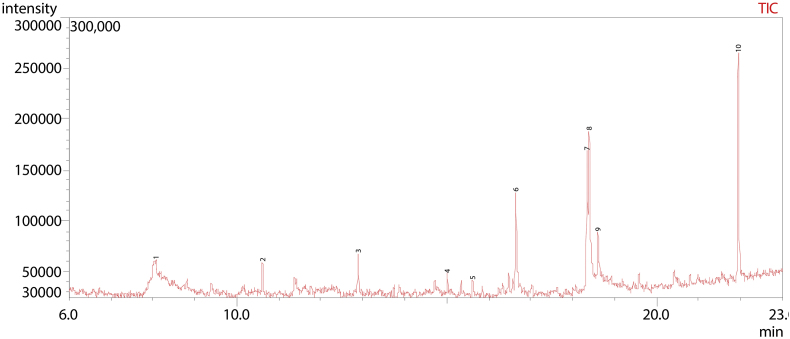
GC-MS chromatogram showing detected compounds in methanolic extract of Ulr-1. Numbers positioned over the peaks indicate the corresponding specific chemical components.

Gas chromatography–mass spectrometry (GC–MS) was employed to analyze the bioactive constituents of two selected fungal extracts: Cer sp-2 and Ulr-1, obtained from red and green algae, respectively. These extracts were chosen for their comparatively strong, broad-spectrum antibacterial activity against all tested bacterial strains, as well as their evident membrane-damaging effects observed by scanning electron microscopy (SEM). The analysis facilitated the identification of major volatile and semi-volatile metabolites that may be associated with the antibacterial and antibiofilm activity and underlying mechanisms of action. The chemical profiles of Cer sp-2 and Ulr-1 extracts were presented in Fig. 7, Fig. 8, Fig. 9, Fig. 10 and Table 13, Table 14, Table 15, Table 16.

**Table 13:** GC-MS-based secondary metabolites identification from Cer sp-2 ethyl acetate extract.

Sample	Peak Nr.	R_T_ (min)	Name of the compound detected	Peak Area %	MW (g/mol)	Molecular Formula
Cer sp-2	1	5.359	1-Decene	3.67	140	C_10_H_20_
	2	8.076	1-Dodecene	6.23	168	C_12_H_24_
	3	10.613	1-Tetradecene	11.09	196	C_14_H_28_
	4	11.952	2,4-Di-*tert*-butylphenol	7.74	206	C_14_H_22_O
	5	12.890	9-Eicosene, (E)-	9.90	280	C_20_H_40_
	6	15.015	Tetradecyl trifluoroacetate	8.03	310	C_16_H_29_F_3_O_2_
	7	16.325	Pyrrolo[1,2-*a*]pyrazine-1,4-dione, hexahydro-	8.49	210	C_11_H_18_N_2_O_2_
	8	16.491	l-Proline, N-valeryl-, heptadecyl ester	9.71	437	C_27_H_51_NO_3_
	9	16.644	l-(+)-Ascorbic acid 2,6-dihexadecanoate	1.95	652	C_38_H_68_O_8_
	10	17.038	9-Tricosene, (Z)-	7.51	322	C_16_H_29_F_3_O_2_
	11	18.072	7-Octadecenoic acid, methyl ester	6.18	296	C_19_H_36_O_2_
	12	18.387	cis-Vaccenic acid	7.66	282	C_18_H_34_O_2_
	13	18.948	1-Nonadecene	3.13	266	C_19_H_38_
	14	20.686	Hexanedioic acid, dioctyl ester	8.73	370	C_22_H_42_O_4_

**Table 14:** GC-MS-based secondary metabolites identification from Cer sp-2 methanolic extract.

Sample	Peak Nr.	R_T_ (min)	Name of the compound detected	Peak Area %	MW (g/mol)	Molecular Formula
Cer sp-2	1	10.614	1-Tetradecene	2.21	196	C_14_H_28_
	2	11.429	Dimethyl(bis[(2Z)-pent-2-en-1-yloxy]) silane	5.39	228	C_12_H_24_O_2_Si
	3	12.890	Trifluoroacetic acid, n-tridecyl ester	2.44	296	C_15_H_27_F_3_O_2_
	4	15.015	1-Nonadecene	1.44	266	C_19_H_38_
	5	16.344	Hexadecanoic acid, methyl ester	3.19	270	C_17_H_34_O_2_
	6	16.646	l-(+)-Ascorbic acid 2,6-dihexadecanoate	10.79	652	C_38_H_68_O_8_
	7	18.013	9,12-Octadecadienoic acid (Z,Z)-, methyl ester	4.50	294	C_19_H_34_O_2_
	8	18.071	9-Octadecenoic acid (Z)-, methyl ester	2.35	296	C_19_H_36_O_2_
	9	18.345	Linoelaidic acid	23.54	280	C_18_H_32_O_2_
	10	18.393	14-Pentadecenoic acid	28.78	240	C_15_H_28_O_2_
	11	18.602	Octadecanoic acid	6.00	284	C_18_H_36_O_2_
	12	21.756	Hexadecanoic acid, 2-hydroxy-1-(hydroxymethyl) ethyl ester	1.74	330	C_19_H_38_O_4_
	13	21.946	Diisooctyl phthalate	3.49	390	C_24_H_38_O_4_
	14	23.486	Butyl 9,12-octadecadienoate	4.15	336	C_22_H_40_O_2_

**Table 15:** GC-MS-based secondary metabolites identification from Ulr-1 ethyl acetate extract.

Sample	Peak Nr.	R_T_ (min)	Name of the compound detected	Peak Area %	MW (g/mol)	Molecular Formula
Ulr-1	1	12.070	l-Glutamic acid, 5-*O*-benzyl-1-*O*-ethyl(ester)	1.43	265	C_14_H_19_NO_4_
	2	12.371	d-Mannitol, 1,4-anhydro-	3.33	164	C_6_H_12_O_5_
	3	12.883	E−14-Hexadecenal	0.28	238	C_16_H_30_O
	4	15.006	Z-5-Nonadecene	0.23	266	C_19_H_38_
	5	15.839	9-Octadecanone	0.14	268	C_18_H_36_O
	6	16.332	Hexadecanoic acid, methyl ester	0.25	270	C_17_H_34_O_2_
	7	16.466	Palmitoleic acid	0.54	254	C_16_H_30_O_2_
	8	16.647	l-(+)-Ascorbic acid 2,6-dihexadecanoate	5.18	652	C_38_H_68_O_8_
	9	17.004	Hexadecanoic acid, ethyl ester	0.30	284	C_18_H_36_O_2_
	10	18.006	8,11-Octadecadienoic acid, methyl ester	0.30	294	C_19_H_34_O_2_
	11	18.062	9-Octadecenoic acid (Z)-, methyl ester	0.20	296	C_19_H_36_O_2_
	12	18.358	Linoelaidic acid	18.06	280	C_18_H_32_O_2_
	13	18.406	9-Octadecenoic acid, (E)-	9.95	282	C_18_H_34_O_2_
	14	18.598	Octadecanoic acid	1.43	284	C_18_H_36_O_2_
	15	18.633	trans,*trans*-9,12-Octadecadienoic acid, propyl ester	1.20	322	C_21_H_38_O_2_
	16	18.690	(E)-9-Octadecenoic acid ethyl ester	0.29	310	C_20_H_38_O_2_
	17	19.585	Hexadecanal, 2-methyl-	0.48	254	C_17_H_34_O
	18	19.754	3-Cyclopentylpropionic acid, 2-dimethylaminoethyl ester	1.51	213	C_12_H_23_NO_2_
	19	21.265	Fumaric acid, 2-dimethyl aminoethyl nonyl ester	7.92	313	C_17_H_31_NO_4_
	20	21.304	Octanoic acid, 2-dimethylaminoethyl ester	4.89	215	C_12_H_25_NO_2_
	21	21.404	9,12-Octadecadienoyl chloride, (Z,Z)-	1.20	298	C_18_H_31_ClO
	22	21.508	Carbonic acid, 2-dimethyl aminoethyl isobutyl ester	0.96	189	C_9_H_19_NO_3_
	23	21.601	Oleoyl chloride	0.64	300	C_18_H_33_ClO
	24	21.751	Hexadecanoic acid, 2-hydroxy-1-(hydroxymethyl)ethyl ester	4.23	330	C_19_H_38_O_4_
	25	23.494	9,12-Octadecadienoic acid (Z,Z)-, 2,3-dihydroxypropyl ester	28.56	354	C_21_H_38_O_4_
	26	23.760	Octadecanoic acid, 2,3-dihydroxypropyl ester	0.58	358	C_21_H_42_O_4_
	27	24.876	Squalene	0.36	410	C_30_H_50_
	28	26.584	Ergosterol	5.55	396	C_28_H_44_O

**Table 16:** GC-MS-based secondary metabolites identification from Ulr-1 methanolic extract.

Sample	Peak Nr.	R_T_ (min)	Name of the compound detected	Peak Area %	MW (g/mol)	Molecular Formula
Ulr-1	1	8.073	2-Pyrrolidineethanamine, 1-methyl-	8.80	128	C_7_H_16_N_2_
	2	10.613	1-Tridecene	2.04	182	C_13_H_26_
	3	12.891	9-Eicosene, (E)-	3.06	280	C_20_H_40_
	4	15.012	Trifluoroacetic acid, pentadecyl ester	1.42	324	C_17_H_31_F_3_O_2_
	5	15.617	Pyrrolo[1,2-*a*]pyrazine-1,4-dione, hexahydro-	2.08	210	C_11_H_18_N_2_O_2_
	6	16.647	l-(+)-Ascorbic acid 2,6-dihexadecanoate	11.23	652	C_38_H_68_O_8_
	7	18.343	Linoelaidic acid	13.24	280	C_18_H_32_O_2_
	8	18.392	*cis*-10-Heptadecenoic acid	24.55	268	C_17_H_32_O_2_
	9	18.601	Octadecanoic acid	10.51	284	C_18_H_36_O_2_
	10	21.947	Diisooctyl phthalate	23.05	390	C_24_H_38_O_4_

As shown in Fig. 7 and Table 13, the total ion chromatogram (TIC) from GC-MS analysis of Cer sp-2 displayed 14 distinct peaks within a retention time range of 5.36 to 20.69 min, indicating the presence of 14 different volatile and semi-volatile compounds. The major constituents include 1-tetradecene (11.09% at peak 3), 9-eicosene, (E)- (9.90% at peak 5), l-proline, N-valeryl-, heptadecyl ester (9.71% at peak 8), hexanedioic acid, dioctyl ester (8.73% at peak 14), pyrrolo[1,2-*a*]pyrazine-1,4-dione, hexahydro- (8.49% at peak 7), and tetradecyl trifluoroacetate (8.03% at peak 6), suggesting that they were relatively abundant compounds in the extract. Several minor constituents, such as 1-decene (3.67% at peak 1), 1-nonadecene (3.13% at peak 13), and l-(+)-ascorbic acid 2,6-dihexadecanoate (1.95% at peak 9), were present in lower intensities.

Similarly, the GC–MS total ion chromatogram (TIC) of the methanolic extract of *Cer* sp-2 revealed 14 distinct and well-resolved peaks within a retention time range of 10.61–23.49 min, indicating the presence of 14 volatile and semi-volatile secondary metabolites. The extract was mainly composed of 14-pentadecenoic acid (28.78%; peak 10), linoelaidic acid (23.54%; peak 9), and 1-(+)-ascorbic acid 2,6-dihexadecanoate (10.79%; peak 6), which represented the major constituents. Other compounds, including diisooctyl phthalate (3.49%; peak 13), hexadecanoic acid, methyl ester (3.19%; peak 5), trifluoroacetic acid, n-tridecyl ester (2.44%; peak 3), 1-tetradecene (2.21%; peak 1), hexadecenoic acid, 2-hydroxy-1-(hydroxymethyl) ethyl ester (1.74%; peak 12), and 1-nonadecene (1.44%; peak 4) were appeared with minor peaks of lower intensity corresponding to less abundant components in the methanolic extract of Cer sp-2 (Fig. 8 and Table 14).

Based on the total ion chromatogram (TIC) of GC-MS analysis, the ethyl acetate extract of Ulr-1 exhibited a complex profile with 28 peaks distributed over a retention time range of 12.07-26.58 min (Fig. 9 and Table 15). A total of 28 compounds were identified, of which 9,12-octadecadienoic acid (Z,Z)-, 2,3-dihydroxypropyl ester (28.56%) at peak 25, linoelaidic acid (18.06%) at peak 12, and 9-octadecenoic acid, (E)- (9.95%) at peak 13 dominated the extract. Likewise, fumaric acid, 2-dimethyl aminoethyl nonyl ester (7.92%) at peak 19, ergosterol (5.55%) at peak 28, l-(+)-ascorbic acid 2,6-dihexadecanoate (5.18%) at peak 8, and octanoic acid, 2-dimethylaminoethyl ester (4.89%) at peak 20 were also detected as prominent constituents, apart from other minor components such as hexadecanoic acid, 2-hydroxy-1-(hydroxymethyl) ethyl ester (4.23%) at peak 24, d-mannitol, 1,4-anhydro- (3.33%) at peak 2, and 3-cyclopentylpropionic acid, 2-dimethylaminoethyl ester (1.51%) at peak 18 in the extract.

The chemical profile of methanolic Ulr-1 extract was analyzed using GC-MS, and the total ion chromatogram (TIC) revealed a chemically diverse composition with well-resolved peaks eluting over a retention time range of 8.07-21.95 min. A total of 10 compounds were tentatively detected. The major constituents of the extract were *cis*-10-heptadecenoic acid (24.55%) at peak 8, diisooctyl phthalate (23.05%) at peak 10, linoelaidic acid (13.24%) at peak 7, l-(+)-ascorbic acid 2,6-dihexadecanoate (11.23%) at peak 6, octadecanoic acid (10.51%) at peak 9, and 2-pyrrolidineethanamine, 1-methyl- (8.80%) at peak 1. In addition, several minor constituents such as 9-eicosene, (E)- (3.06%) at peak 3, pyrrolo[1,2-*a*]pyrazine-1,4-dione, hexahydro- (2.08%) at peak 5, 1-tridecene (2.04%) at peak 2, and trifluoroacetic acid, pentadecyl ester (1.42%) at peak 4 were detected as minor peaks. These lower intensity peaks indicate the lesser abundance of these compounds in the methanolic extract of Ulr-1 (Fig. 10 and Table 16).

## 4 Discussion

Marine-derived fungi, particularly those inhabiting the surface of macroalgae, have emerged as prolific sources of several bioactive metabolites. Among them, epiphytic fungi living externally on algal thalli play a crucial ecological role in protecting their hosts from microbial colonization, predation, and fouling through the secretion of defensive compounds. These chemical defenses, evolved in competitive marine environments, often exhibit potent antibacterial and anti-biofilm activities with promising implications for human health applications.^55^ Despite their capability, marine fungi remain far less studied than their terrestrial counterparts, offering a vast reservoir of untapped biodiversity and bioactive potential.^56^ While many studies worldwide have attempted to demonstrate the great potential of marine-origin fungi, there is no sufficient information regarding the significance of seaweed-associated epiphytic fungal flora along the Kenyan coast. A more comprehensive understanding is required, as the bioactivity and chemical profiles of fungi associated with marine hosts vary depending on geographical region and host species.^57^ This makes their study highly relevant for discovering novel compounds that are often absent or less abundant in other microbial sources. The present study provides evidence that fungal epiphytes associated with red (Rhodophyta) and green (Chlorophyta) algae from the Kenyan coast harbor species capable of producing metabolites with significant antibacterial and anti-biofilm potential.

Morphological characterization, supported by ITS-based molecular phylogeny, confirmed that the fungal isolates belonged to five genera: *Fusarium*, *Aspergillus*, *Alternaria*, *Penicillium*, and *Talaromyces*—all of which are known for producing antimicrobial, cytotoxic, and antifungal compounds.^58^ Several isolates clustered with reference strains previously reported to synthesize metabolites such as terreic acid, questin, and various polyketides, thereby strengthening the likelihood that the observed bioactivities in this study are attributable to chemically diverse secondary metabolites encoded within their biosynthetic gene clusters.^59^ This was also similar to what has been described in other culture-dependent studies on marine fungi associated with red and green algae.^60^

Despite the fact that the internal transcribed spacer (ITS) region is widely accepted as the universal DNA barcode for fungi, its discriminatory power is often insufficient for robust species-level identification in several taxonomic groups.^61^ In the present study, sequence similarity and phylogenetic analyses based on ITS data enabled preliminary placement of the isolates; however, some clades (Acs-2, Hym-1, Ula-1, and Ulr-1) were supported by low to moderate bootstrap values with reference sequences. Consequently, the species-level assignments reported here should be regarded as indicative of the closest phylogenetic affinities rather than definitive identifications.^62^ A more robust taxonomic framework would require multi-locus sequence analysis incorporating additional genetic markers such as translation elongation factor 1-alpha (TEF1-α), β-tubulin, or large subunit (LSU) rDNA regions, which have been shown to significantly improve species resolution in fungi.^63^ Future studies integrating multi-locus approaches, together with expanded sampling, will be essential for achieving robust species delimitation and a more comprehensive understanding of fungal epiphyte diversity along the coast.

The antibacterial activity of fungal epiphytes was primarily assessed using the agar plug diffusion method, an approach ideal for detecting the secreted antibacterial compounds directly from agar plugs (Table 2, Table 3). Afterward, the disc diffusion assay was employed to evaluate the antibacterial activity of both fermentation broths and mycelial extracts, a method widely acknowledged as a better choice of precision for secondary screening.^64^ Marine fungal epiphytes naturally secrete their bioactive compounds into the surrounding medium as chemical defenses against microbial competitors and predators. Fungal extracts investigated in this study showed notable antibacterial activity, with both ethyl acetate and methanolic fractions inhibiting bacterial growth. This clearly indicates that the bioactive metabolites were present in both extracellular and intracellular compartments. With the exception of Hym-1, which showed antibacterial activity against *E. cloacae* only in its methanolic extract, nearly all other isolates exhibited relatively stronger inhibitory activities in their ethyl acetate extracts against the tested pathogens. These findings concur with previous studies reporting that solvent polarity critically influences the recovery of bioactive metabolites.^65^ Isolates Acs-2, Cer sp-2, Ula-1, Ulr-1, and Ulr-18 consistently displayed better activities, recording some of the lowest MIC (0.039 mg/mL) and MBC (0.156 mg/mL) values, particularly against Gram-negative bacteria (*P. aeruginosa*) as well as the Gram-positive pathogens (*S. aureus* and *E. faecium*). The MIC and MBC results of the study further reveal that the ethyl acetate and methanol extracts are effective antibacterial agents individually, but they are even more effective when acting dually combined.

With the exception of *P*. *aeruginosa,* Gram-positive bacteria generally manifested a stronger response to the fungal extracts than their Gram-negative counterparts, in agreement with previous studies indicating that Gram-positive bacteria are typically more susceptible to fungal-derived metabolites.^66^ This condition is largely caused by the difference in cell wall structure of these two classes of bacteria that serves as a permeability barrier to impede the penetration of toxic substances into the cells. Theoretically, the cell wall of Gram-positive bacteria consists of a thick and porous peptidoglycan layer without the presence of outer membrane, which causes it to become permeable to most antibiotics. On the contrary, the cell wall of Gram-negative bacteria contains more complex substances with an outer membrane layer, thereby conferring greater resistance to most antibacterial agents.^67^

*P. aeruginosa,* a Gram-negative bacterium typically known for its high intrinsic resistance, was exceptionally susceptible to several of the fungal extracts. This unusual sensitivity could be explained by the presence of specific fungal metabolites that disrupt the integrity of its outer membrane or interfere with critical physiological processes, such as quorum sensing and efflux pump regulation. Such interference may weaken its protective mechanisms, increasing permeability, and facilitating the entry of active compounds, which could compromise its defenses and render it more susceptible than other Gram-negative bacteria.^68^ Interestingly, most of the extracts inhibited the growth of drug-resistant ESKAPE pathogens, where the commercial antibiotic disc (positive control) showed no measurable inhibitory activity.^25^^,^^69^ However, both the fungal extracts and conventional antibiotics were effective against the standard (reference) strains tested in the study. This observation underscores the potential of epiphytic fungal extracts as alternative leads against the most clinically challenging bacterial infections.

Previous studies confirmed that fungi associated with marine hosts are good producers of various biological activities and biotechnological applications. For example, a study conducted on *Fusarium equiseti* derived from marine mangroves reported inhibitory activity against drug-resistant *P. aeruginosa* at 12.5 mg/mL.^70^ In the present study, both the ethyl acetate and methanol extracts of Acs-2 (*Fusarium* sp. Acs-2) also demonstrated broad-spectrum inhibition against all the tested pathogens. Similarly, Ula-1 (*Fusarium* sp. Ula-1) displayed robust antibacterial efficacy, consistent with reports on marine *Fusarium solani* 8388, which produced novel polyketides (fusarisolins F–K) showing MIC values as low as 3 μg/mL against Methicillin-resistant *Staphylococcus aureus* (MRSA).^71^ These findings reaffirm *Fusarium* spp. as promising sources of antibacterial metabolites in marine and algal epiphytic environments. Among the extracts assessed, Cer sp-2 (*Aspergillus fumigatus*) exhibited comparatively strong antibacterial effects, with MIC and MBC values of 0.039 mg/mL and 0.156 mg/mL, respectively, as observed in *P. aeruginosa*. This aligns with studies on marine-derived *A. fumigates* H22, which provided 45 secondary metabolites, nearly half showing remarkable antibacterial activity against MRSA.^72^ This further validates the isolate Cer sp-2 pharmaceutical relevance in the future. Next to Cer sp-2, Ulr-1 (*Penicillium* sp. Ulr-1) demonstrated pronounced activity against both Gram-negative and Gram-positive bacteria, consistent with previous reports on marine *P. citrinum* VM6 extracts that produced metabolites with low MIC values against several pathogenic bacteria.^73^ Lastly, Ulr-18 (*Aspergillus aculeatus*) showed antibacterial potential aligning with prior studies on marine *Aspergillus* species, where *A. aculeatinus* (a close relative) produced dimeric tetrahydroxanthones (aculeaxanthones A-E) with inhibitory activities against *B. subtilis*, *S. aureus*, and *H. pylori*.^74^

The mechanisms underlying these activities were further supported by scanning electron microscopy (SEM) analyses, which identified pronounced morphological changes in bacterial cells treated with the fungal extracts. As illustrated in Fig. 4, Fig. 5, S *aureus* underwent significant surface collapse and cellular deformation, whereas *E. cloacae* exhibited membrane rupture and cytoplasmic leakage. These ultrastructural alterations indicate that metabolites derived from the ethyl acetate and methanol (combined) extract of Acs-2, Cer sp-2, Ula-1, Ulr-1, and Ulr-18 isolates act through membrane-targeting mechanisms. In this case, the bioactive constituents within these extracts interact with the outer membrane or cell wall, facilitating the permeation of antimicrobial agents and compromising structural stability. Such disruption most likely leads to a loss of cellular balance and eventual lysis. These observations align with the findings reported by Chatterjee and his colleagues, who demonstrated that the ethyl acetate extract of *A. alternata* AE1 caused severe cell wall degradation, resulting in irreversible cellular damage.^75^

In the current findings, the reduction in Gram-positive and Gram-negative bacterial biofilms by the fungal extracts was significantly different compared to the PBS-treated control (∗p < 0.05, ∗∗P *<* 0.01, ∗∗∗P *<* 0.001, and ∗∗∗∗P *<* 0.0001). Despite this, the crystal violet assay showed relatively low reduction percentages, likely due to the assay measures total biofilm biomass, including viable cells and extracellular matrix, rather than only viable cell reduction. Thus, extracts affecting cell viability, quorum sensing, or matrix synthesis may cause biofilm damage without greatly reducing biomass staining. While all the extracts showed remarkable antimicrobial activities against ESKAPE pathogens, only Acs-2 and Cer sp-2 extracts of fungal epiphytes exhibited good percentage of biofilm reduction. These findings confirmed that good antimicrobial activities of extracts did not correspond with good anti-biofilm formation activities.^76^ The observed weak percentage of biofilm reduction activity of the extracts against the test bacteria could be associated with the ability of the test bacteria to withstand the anti-biofilm effect of the extracts, their inherent resistance to antimicrobial agents, and genetically regulated process.^77^ One of the many possible reasons for the apparent good anti-biofilm activities of the extracts might be attributed to their capability to reduce biofilm sites (e.g., cyclic diguanylate) of the test bacteria. Cyclic diguanylate initiates production of bis-(3′-5′)-cyclic dimeric guanosine monophosphate to facilitate the continuous secretion of adhesive substances which have a direct correlation with the biofilm formation process of the bacteria.^78^

To gain insight into the chemical basis of the morphological alterations observed in scanning electron microscopy (SEM), the ethyl acetate and methanolic extracts of the two representative fungal extracts (Cer sp-2 and Ulr-1) were further analyzed using GC–MS. This approach enabled profiling of the detected metabolites, providing information on retention times (R_T_), relative abundance (peak area), molecular weights, and corresponding molecular formulas, as reflected in the total ion chromatograms (TICs) and detailed in Table 13, Table 14, Table 15, Table 16. The notable antibacterial activity exhibited by these extracts appears to be associated with their chemically heterogeneous composition. In particular, the identified metabolites predominantly included aliphatic hydrocarbons, sterols, fatty acid derivatives, monoglycerides, cyclic dipeptides, phenolics, amino-substituted esters, and low-molecular-weight polar metabolites, all of which are known to contribute to antimicrobial properties.^79^

A significant portion of the extracts in Cer sp-2 and Ulr-1 is composed of unsaturated fatty acids, including linoelaidic acid and oleic acid. These molecules are known to act as natural detergents that increase the permeability of bacterial cell membranes. Furthermore, the presence of lipophilic phenolic compound (2,4-di-*tert*-butylphenol) in the Cer sp-2 ethyl acetate extract has been reported to inhibit bacterial growth by inducing intracellular leakage and inhibiting essential enzyme systems.^80^ The detection of pyrrolo[1,2-*a*]pyrazine-1,4-dione, hexahydro (a diketopiperazine) in the ethyl acetate extract of Cer sp-2 and the methanolic extract of Ulr-1 further strengthens the antimicrobial profile, as this class of secondary metabolites often interferes with bacterial quorum sensing and protein synthesis. The chemical composition highly correlates with the morphological changes observed in the scanning electron microscopy (SEM) analysis. The SEM micrographs typically show significant cell wall distortion, membrane lysis, and “shriveling” of the treated bacterial cells.^81^ These structural damages can be attributed to the high concentration of surfactants identified in the GC-MS reports. The presence of monoglycerides (9,12-Octadecadienoic acid (Z,Z)-, 2,3-dihydroxypropyl ester and fatty acid esters in Ulr-1 extract suggests a mechanism where these amphiphilic molecules intercalate into the bacterial lipid bilayer.^82^ This leads to the pore formation observed under SEM. The reactive nature of acyl chlorides and trifluoroacetate esters identified in the Cer sp-2 and Ulr-1 extracts may contribute to the irreversible damage of the peptidoglycan layer, resulting in the total loss of cellular integrity visible in the SEM images. The abundance of alkenes and sterol-like ergosterol in the fungal extracts may also interfere with the lipid organization of the target bacteria, particularly in Gram-positive strains, facilitating the membrane collapse seen in the results of SEM analysis.^83^^,^^84^ Thus, the GC-MS profiles of Cer sp-2 and Ulr-1 provided a robust biochemical foundation for the antimicrobial activity demonstrated via SEM imaging and standard antibacterial evaluations.

## 5 Conclusions and recommendations

To the best of our knowledge, this study presents the first report on cultivable fungal epiphytes associated with red and green macroalgae from a single intertidal site of the Kenyan coast, sampled during a single collection period. Morphological and ITS-based identification confirmed that the isolates belong to the genera: *Fusarium*, *Alternaria*, *Aspergillus*, *Talaromyces*, and *Penicillium*. Both methanolic and ethyl acetate extracts exhibited remarkable antibacterial activity, with the latter yielding relatively stronger bioactive compounds. Notably, fungal extracts were unique in that they were effective against multidrug-resistant ESKAPE pathogens, even in cases where conventional antibiotic discs showed no measurable inhibition, witnessing their promise as alternative leads for combating antimicrobial resistance. Among them, Acs-2 and Ulr-1 particularly showed low MIC and MBC values, indicating prospective candidates for drug development. The combined extracts demonstrated better antibacterial activity compared to the individual ethyl acetate and methanolic fractions, as clearly supported by the MIC and MBC values for each fungal isolate. SEM analyses revealed that their metabolites disrupt bacterial membranes, causing ultrastructural damage and cell death. This finding also showed that selected extracts effectively reduced the biofilm at sub-MIC values, although their ability to eradicate mature biofilms was to some extent limited. Generally, the current study underscores the potential of marine-derived epiphytic fungi as a valuable source of novel antibacterial agents. Further studies are needed to integrate compound purification and structural elucidation with advanced membrane disruption assays to identify potential therapeutic candidates and validate the underlying mechanism of action.

### 5.1 Limitations of the study

The findings were not intended to represent the complete fungal epiphyte diversity across the Kenyan coastline. More comprehensive ecological conclusions will therefore require expanded studies incorporating multiple sites, seasonal sampling, and culture-independent approaches. Future work should also consider incorporating multi-locus phylogenetic approaches to improve species-level resolution of fungal epiphytes. Regarding the methodology, the term “combined extracts” refers to the co-application of ethyl acetate and methanolic extracts. No formal synergy assessment (checkerboard assay or FICI analysis) was conducted; therefore, the observed effects should not be interpreted as confirmed synergistic interactions.

## CRediT authorship contribution statement

**Aragaw Zemene Sendekie:** Writing – review & editing, Writing – original draft, Visualization, Validation, Software, Resources, Project administration, Methodology, Investigation, Funding acquisition, Formal analysis, Data curation, Conceptualization. **Kimang'a Andrew Nyerere:** Writing – review & editing, Visualization, Validation, Supervision, Resources, Project administration, Methodology, Investigation, Funding acquisition, Data curation, Conceptualization. **Purity Kinya Kaaria:** Writing – review & editing, Visualization, Validation, Supervision, Resources, Project administration, Methodology, Investigation, Funding acquisition, Data curation, Conceptualization.

## Availability of data:

All data generated or analyzed during the study will be available upon request. Nucleotide sequences with their accession numbers have been deposited in GenBank (Link: https://www.ncbi.nlm.nih.gov/nuccore/?term=PV358366:PV358383[accn]).

## Funding

The study was supported by the 10.13039/501100011951African Union under its scholarship program through the 10.13039/501100022253Pan African University Institute for Basic Sciences, Technology, and Innovation (10.13039/501100024784PAUSTI), Kenya.

## Declaration of competing interest

The authors declare that they have no known competing financial interests or personal relationships that could have appeared to influence the work reported in this paper.
